# In-situ generation of large numbers of genetic combinations for metabolic reprogramming via CRISPR-guided base editing

**DOI:** 10.1038/s41467-021-21003-y

**Published:** 2021-01-29

**Authors:** Yu Wang, Haijiao Cheng, Yang Liu, Ye Liu, Xiao Wen, Kun Zhang, Xiaomeng Ni, Ning Gao, Liwen Fan, Zhihui Zhang, Jiao Liu, Jiuzhou Chen, Lixian Wang, Yanmei Guo, Ping Zheng, Meng Wang, Jibin Sun, Yanhe Ma

**Affiliations:** 1 Key Laboratory of Systems Microbial Biotechnology, Tianjin Institute of Industrial Biotechnology, Chinese Academy of Sciences, Tianjin, China; 2 National Technology Innovation Center of Synthetic Biology, Tianjin, China; 3 University of Chinese Academy of Sciences, Beijing, China; 4 School of Life Sciences, University of Science and Technology of China, Hefei, China

**Keywords:** Microbiology techniques, Metabolic engineering, Applied microbiology, CRISPR-Cas9 genome editing

## Abstract

Reprogramming complex cellular metabolism requires simultaneous regulation of multigene expression. Ex-situ cloning-based methods are commonly used, but the target gene number and combinatorial library size are severely limited by cloning and transformation efficiencies. In-situ methods such as multiplex automated genome engineering (MAGE) depends on high-efficiency transformation and incorporation of heterologous DNA donors, which are limited to few microorganisms. Here, we describe a Base Editor-Targeted and Template-free Expression Regulation (BETTER) method for simultaneously diversifying multigene expression. BETTER repurposes CRISPR-guided base editors and in-situ generates large numbers of genetic combinations of diverse ribosome binding sites, 5’ untranslated regions, or promoters, without library construction, transformation, and incorporation of DNA donors. We apply BETTER to simultaneously regulate expression of up to ten genes in industrial and model microorganisms *Corynebacterium glutamicum* and *Bacillus subtilis*. Variants with improved xylose catabolism, glycerol catabolism, or lycopene biosynthesis are respectively obtained. This technology will be useful for large-scale fine-tuning of multigene expression in both genetically tractable and intractable microorganisms.

## Abstract (web summary)

To obtain optimal yield and productivity in bioproduction, expression of pathway genes must be appropriately coordinated. Here, the authors report repurposing of base editors for simultaneous regulation of multiple gene expression and demonstrate its application in industrially important and model microorganisms.

## Introduction

One of the key research areas in synthetic biology is to reprogram metabolic pathways, which involve multiple indigenous and/or heterologous genes, for metabolite overproduction^1^. In order to obtain optimal yield and productivity, the expression of pathway genes must be appropriately balanced to avoid metabolic bottlenecks or burdens^2,3^. Although cloning methods such as Gibson assembly^4^ allow ex situ construction of combinatorial libraries for multiple genes controlled by diverse promoters or RBSs, the reported maximum number of target genes is limited to five with a maximum pool size of 3,125 (5^5^, five genes × five promoters)^5^. With the advent of multiplex automated genome engineering (MAGE)^6^ and eukaryotic MAGE^7^, the incorporation of synthetic DNAs into chromosomes was employed to generate in situ multisite genome modifications. Through the repeated introduction of synthetic DNAs for several cycles, a large population of variants can be generated for pathway diversification^6,7^. Clustered regularly interspaced short palindromic repeats (CRISPR)-based methods such as CRMAGE^8^ and CFPO^9^ improve the recombineering efficiency of MAGE by double-stranded DNA breaks (DSBs)-mediated counterselection. However, synthesis of complex DNA template pools, expression of multiple guide-RNAs (gRNAs), and cellular toxicity of DSBs limit the number of targetable genes. These limitations make the existing methods only viable for a limited number of microorganisms with high DNA transformation and incorporation efficiencies, namely *Escherichia coli*^2,6,8,9^ and *Saccharomyces cerevisiae*^3,5,7^. New approaches are needed to break the barrier of low-efficiency DNA cloning, transformation, and incorporation for simultaneous regulation of multiple genes.

Base editing is a new genome editing technology that uses fusions between a catalytically impaired CRISPR-associated (Cas) nuclease and a nucleobase deaminase to directly install nucleobase transitions in genomic DNA without generating DSBs, requiring a DNA donor template, or relying on homologous recombination^10,11^. Two classes of DNA base editor have been described, cytosine base editors (CBEs) for C∙G base pair into T∙A base pair conversion (Target-AID series and BE series)^12,13^, and adenine base editors (ABEs) for A∙T base pair into G∙C base pair conversion^14^. Since their invention, base editors have been quickly adopted for correcting pathogenic point mutations, gene silencing, protein engineering, etc.^15–17^.

Here, we report repurposing of base editors for simultaneous regulation of multigene expression (a strategy we have termed BETTER for Base Editor-Targeted and Template-free Expression Regulation). BETTER is designed to create complex libraries of genetic variants on multiple translations or transcription elements such as ribosome binding site (RBS) in situ via base editing, which forms large numbers of genetic combinations for multiple genes expressed at diverse levels. We successfully apply BETTER in industrially important and model microorganisms *Corynebacterium glutamicum* and *Bacillus subtilis*, whose genetic manipulations suffer from low transformation and recombination efficiencies for large genetic combinatorial library creation. Expression of up to ten genes is simultaneously tuned for enhanced substrate uptake or natural product biosynthesis.

## Results

### Design of the BETTER method

**Fig. 1:**
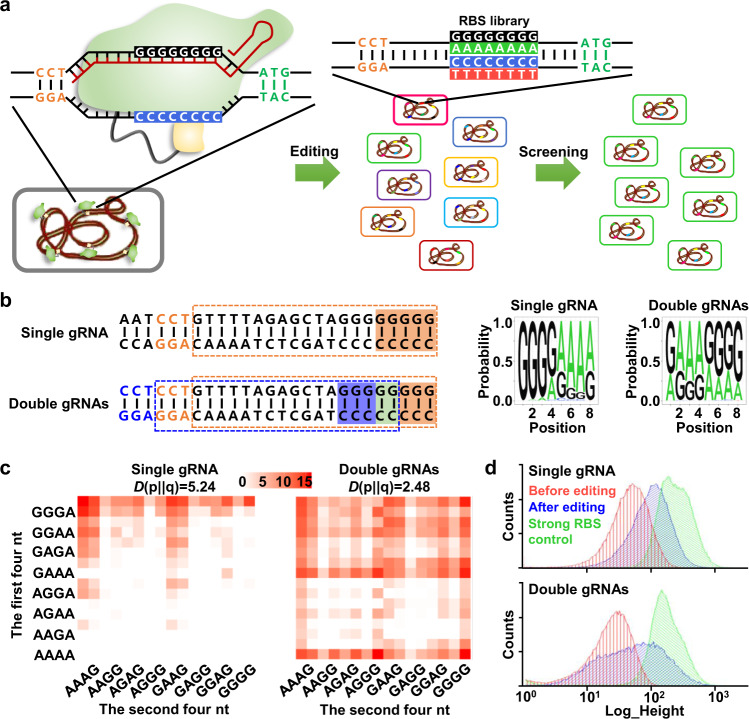
General workflow and validation of BETTER for generating genetic combinations and diversifying gene expression. **a** BETTER repurposes CRISPR-guided nCas9-cytidine deaminase fusion to generate G/A-rich RBS libraries from tailored starting RBSs with eight consecutive Gs and produce variant cells with different phenotypes. **b** A single gRNA leads to predominant editing of the five Gs distal to PAM while two interlaced gRNAs lead to less biased editing of eight Gs. A set of PAM, gRNA, and editing window is shown in the same color. The two Gs shaded in green are covered by two editing windows (blue and orange). Three colonies were used for base editing and the cells were mixed with an equal proportion before extraction of genomic DNAs, PCR amplification, and NGS. **c** The matrixes show the coverage and discreteness of 256 G/A-containing RBS variants in the RBS libraries generated by BETTER with single or double gRNAs. KL divergence was used to evaluate the discreteness of genetic combinations. Scale bar represents log_2_(relative proportion). **d** Strength of the RBS libraries generated by BETTER was analyzed for GFP expression by flow cytometry. A strong RBS (GAAAGGAG) was used as a control. Source data underlying Fig. 1c are provided as a Source Data file.

RBS is a G/A-rich nucleotide sequence upstream of the start codon of an mRNA transcript that directly tunes gene expression due to sequence-dependent ribosome recruitment during initiation of protein translation^6^. Starting from a tailored RBS with eight consecutive Gs, BETTER is programmed to generate a G/A-rich RBS library for a target gene via C to T transition on the complementary strand (Fig. 1a). The maximum RBS library size for each target gene is 256 (G/A, 2^8^) considering the major C∙G → T∙A transition and can reach 65,536 (G/A/C/T, 4^8^) considering the minor C∙G → G∙C and C∙G → A∙T by-products of CBEs^12,13^. Taking advantage of the superior multiplex editing capability of base editors^18,19^, BETTER can target dozens of genes simultaneously and produce large numbers of genetic combinations with diverse expression profiles of target genes.

### Diversifying GFP expression by BETTER

BETTER was first used to diversify the expression of a green fluorescence protein (GFP) reporter in *C. glutamicum*, a major workhorse in industrial biotechnology used for the production of approximately 70 natural and nonnatural compounds^20^. The DNA encoding GFP was integrated into the chromosome with a constitutive promoter and the tailored GGGGGGGG RBS (Supplementary Fig. 1). Previously constructed Target-AID base editing tool plasmids^18^ were engineered to express a Cas9 nickase (nCas9)-cytidine deaminase fusion and an RBS-targeting gRNA. After plasmid transformation and induced base editing, targeted next-generation sequencing (NGS) was used to examine the outcomes of base editing. For all NGS analyses, the genomic DNAs of ~10^9^ cells were extracted and used as templates to amplify the base editing region. The amplicon was sequenced and approximately 100,000 reads per sample were analyzed. The sequencing data revealed an editing bias toward the five Gs distal to protospacer-adjacent motif (PAM) (Fig. 1b), which is consistent with the reported five-base editing window of cytidine base editors^13^. To cover the Gs proximal to PAM and obtain a more evenly distributed RBS library, a second NGG PAM was inserted next to the first one and a second gRNA interlaced with the first one was additionally expressed (Fig. 1b). This modification improved the type of generated RBS variants from 94 to 202 out of 256 G/A-containing combinations. A Kullback–Leible (KL) divergence analysis was used to quantify the discreteness of RBS variants generated by BETTER and the lower KL divergence suggested distribution of RBS variants tended to be more even (Fig. 1c and Supplementary Data 1). In the case of calculating G/A/C/T-containing combinations, this modification improved the type of RBS variants from 527 to 970 (Supplementary Data 1). The strength of generated RBS variants spanned nearly three orders of magnitude in terms of GFP fluorescence intensity (Fig. 1d), which is comparable to those of previous plasmid-borne promoter libraries or CRISPR activation/interference devices and large enough for reprogramming cellular metabolism^5,21,22^.

### Triple-genes regulation for reprogramming xylose catabolism

**Fig. 2:**
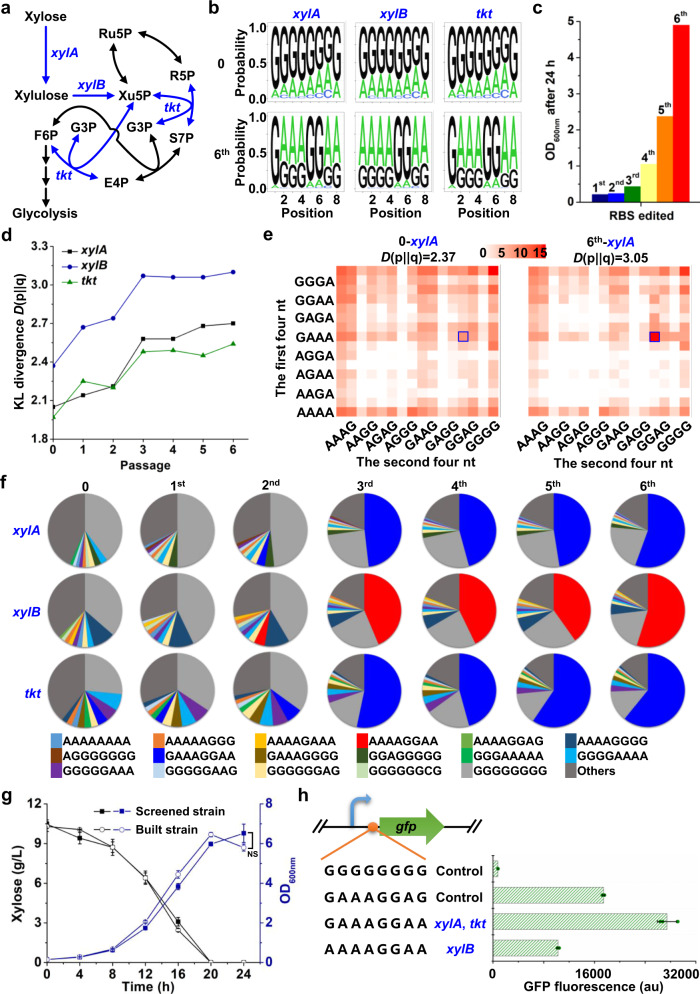
Reprogramming xylose catabolism by BETTER. **a** Xylose catabolism pathway. Three target genes and the corresponding reactions are highlighted in blue. E4P erythrose 4-phosphate, F6P fructose 6-phosphate, G3P glyceraldehyde 3-phosphate, R5P ribose 5-phosphate, Ru5P ribulose 5-phosphate, S7P sedoheptulose 7-phosphate. **b** Components of RBS libraries before (0) and after six passages (sixth) of serial cultivation in xylose. Three colonies were used for base editing and the cells were mixed with an equal proportion before extraction of genomic DNAs, PCR amplification, and NGS. **c** Serial cultivation for screening genetic combinations that favor growth on xylose. The numbers above columns represent the passages of serial cultivation. **d** Discreteness change of genetic combinations during serial cultivation. KL divergence was used to evaluate the discreteness of genetic combinations. **e** The matrixes show the coverage and discreteness of 256 G/A-containing RBS variants in the RBS libraries generated by BETTER before (0) and after six passages (sixth) of serial cultivation in xylose. Scale bar represents log_2_(relative proportion). Blue squares represent the enriched GAAAGGAA RBS_*xylA*_. **f** RBS variants with the largest ten proportions for each passage of serial cultivation. **g** Xylose utilization by a screened strain from the sixth passage and a rationally built strain with the same *xylA*, *xylB*, and *tkt* RBSs as the screened strain. Values and error bars reflect the mean ± s.d. of three biological replicates (*n* = 3). Statistical evaluation (*P* value) was performed by a two-sided *t* test. NS nonsignificant (*P* ≥ 0.05), *n* = 3. **h** Strength of RBSs enriched during serial cultivation in xylose using a chromosomal GFP reporter. The tailored RBS (GGGGGGGG) and a strong RBS (GAAAGGAG) were used as controls. Values and error bars reflect the mean ± s.d. of three biological replicates (*n* = 3). Source data underlying Fig. 2c–h are provided as a Source Data file.

We next used BETTER to optimize the pathway for xylose catabolism, which is an industrially important phenotype for lignocellulose conversion^23^. A heterologous *xylA* gene from *E. coli* was first integrated into *C. glutamicum* chromosome with a constitutive promoter and the tailored GGGGGGGG RBS to complete the pathway (Fig. 2a and Supplementary Fig. 2). Another two essential genes for xylose utilization, *xylB* and *tkt*, were selected as targets of BETTER and equipped with the same tailored RBS, which allowed the three targets to share the same gRNA. Prior to inducing the expression of CRISPR-guided cytidine deaminase, leaky base editing events severer than the single-site editing at RBS_*gfp*_ were observed, which might be due to the increase in editing sites (Supplementary Fig. 3). To reduce the leaky expression of the base editor and biased editing of some nucleotides, we used a weaker RBS to weaken the translation of CRISPR-guided cytidine deaminase (Supplementary Fig. 4). This modification led to an overall moderate and less biased editing of eight Gs (Fig. 2b and Supplementary Fig. 3). Targeted sequencing revealed the G/A/C/T-containing types of RBS_*xylA*_, RBS_*xylB*_, and RBS_*tkt*_ variants were 3,297 (including 213 G/A-containing types), 5,778 (including 239 G/A-containing types), and 3,464 (including 229 G/A containing types), making the estimated theoretically maximum library complexity of 6 × 10^10^ (3,297 × 5,778 × 3,464) (Supplementary Data 2).

After curing of tool plasmids, the edited cell mixture was serially cultivated to enrich fast-growing variants using xylose as a sole carbon source. The starting strain without base editing was also serially cultivated as a control. After six passages of serial cultivation, the growth of the edited cell mixture on xylose was significantly improved (Fig. 2c). However, the unedited starting strain did not grow on xylose at all (Supplementary Fig. 5), which was possible due to the low-level expression of xylose utilizing enzymes with the tailored GGGGGGGG RBS (Supplementary Fig. 6). For the edited cell mixture, cells were collected from each passage and RBSs for target genes were analyzed by NGS. During serial cultivation, the KL divergence values for RBS variants increased, implying the increasing maldistribution of RBS variants and enrichment of certain RBS variants (Fig. 2d, e). Detailed analysis suggested that RBS_*xylA*_ GAAAGGAA, RBS_*xylB*_ AAAAGGAA, and RBS_*tkt*_ GAAAGGAA became the dominant RBS combinations after six passages of serial cultivation (Fig. 2b, Supplementary Fig. 7, and Supplementary Data 3). The three RBS variants were enriched by 209-, 52-, and 13-folds, respectively, and accounted for over half of RBS variants in the sixth passage (Fig. 2f and Supplementary Data 3). Four fast-growing single colonies picked from the sixth passage all had the aforementioned RBS combinations. The specific growth rate and xylose uptake rate of an enriched strain reached 0.26 h^−1^ and 7.3 mmol/gCDW h, respectively (Fig. 2g), which is above the reported fastest xylose uptake rate of *C. glutamicum* (5.7 mmol/gCDW h)^24^.

To exclude the effects of other unexpected mutations accumulated during serial cultivation, the original GGGGGGGG RBSs of *xylA*, *xylB*, and *tkt* was replaced with the enriched RBS combinations in the starting strain, which produced a strain with the same xylose utilization capability (Fig. 2g). The results suggest that improved xylose utilization is solely due to the RBS changes. Using a chromosomal GFP reporter, the enriched RBSs (GAAAGGAA and AAAAGGAA) were tested for their translation efficiency. The GAAAGGAA and AAAAGGAA RBSs exhibited 35- and 13-fold higher translation efficiencies relative to the original GGGGGGGG RBS. When compared with a commonly used strong RBS (GAAAGGAG), the GAAAGGAA and AAAAGGAA RBSs exhibited 157% and 59% strength (Fig. 2h). Since the 5′ region of coding sequence (CDS) also affects gene expression^25,26^, we directly measured activities of xylose utilizing enzymes in the starting strain with GGGGGGGG RBS and the screened strain with edited RBSs. The activities of XylA, XylB, ant Tkt in crude cell extracts increased by 7.0-, 5.8-, and 10.4-fold due to the RBS changes (Supplementary Fig. 6), which was intuitive and consistent with previous findings that xylose utilization benefited from the elevated expression of xylose catabolic genes^9^. The results also suggest that one RBS can lead to different expression levels for different target genes (eg. *gfp* and xylose catabolic genes) possibly due to the 5′ region of CDS. In this case, the BETTER method is highly preferred for optimizing gene expression since it helps to directly screen the optimal RBS for a target gene.

### Decuple-genes regulation for reprogramming lycopene biosynthesis

**Fig. 3:**
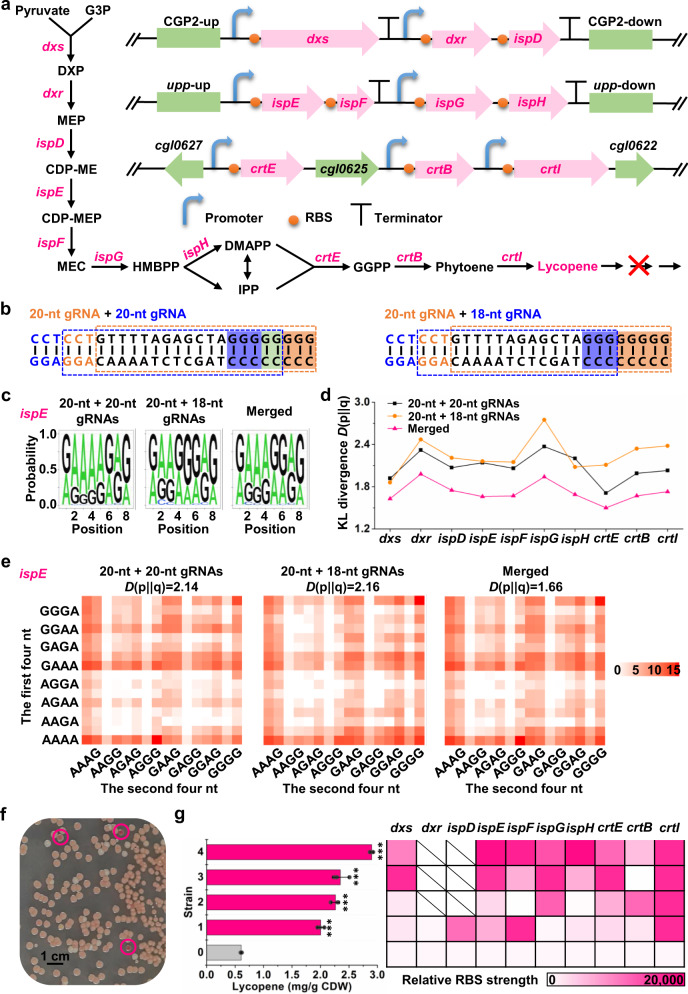
Reprogramming lycopene biosynthesis by BETTER. **a** Lycopene biosynthesis pathway and three artificial clusters equipped with constitutive promoters and tailored GGGGGGGG RBSs. Ten target genes are highlighted in red. CDP-ME 4-diphosphocytidyl-2-C-methyl-d-erythritol, CDP-MEP 4-diphosphocytidyl-2C-methyl-d-erythritol-2-phosphate, DMAPP dimethylallyl diphosphate, FPP farnesyl diphosphate, GGPP geranylgeranyl pyrophosphate, HMBPP (*E*)-4-hydroxy-3-methylbut-2-enyl-diphosphate, IPP isopentenyl diphosphate, MEC 2C-methyl-d-erythritol-2,4-cyclodiphosphate, MEP 2-C-methyl-d-erythritol-4-phosphate. **b** Schematic illustration of two sets of double-gRNAs combinations and corresponding editing windows. The two Gs shaded in green are covered by two editing windows (blue and orange). **c** Components of RBS libraries of *dxs* gene generated by BETTER with the two sets of double-gRNAs combinations. Merged represents the sum of RBS variants generated by the aforementioned two editing events. Three colonies were used for base editing and the cells were mixed with an equal proportion before extraction of genomic DNAs, PCR amplification, and NGS. **d** Discreteness change of genetic combinations of RBS_*ispE*_ generated by BETTER with the two sets of double-gRNAs combinations. KL divergence was used to evaluate the discreteness of genetic combinations. **e** The matrixes show the coverage and discreteness of 256 G/A-containing RBS_*ispE*_ variants in the RBS libraries generated by BETTER with the two sets of double-gRNAs combinations. Scale bar represents log_2_(relative proportion). **f** Screening lycopene overproduction cells from the libraries generated by BETTER. **g** Lycopene production and RBS strength assay of the ancestral strain (strain 0) and four screened strains with intense red pigmentation (strains 1-4). RBS strength was determined using a chromosomal GFP reporter. Scale bar represents GFP fluorescence normalized with OD_600 nm_. Values and error bars reflect the mean ± s.d. of three biological replicates (*n* = 3). Statistical evaluation (*P* value) of the comparison between each screened strain and the ancestral strain was performed by a two-sided *t* test. ***P < 0.001, *n* = 3. Source data underlying Fig. 3d, e, g are provided as a Source Data file.

To demonstrate an application of BETTER in optimizing more complex metabolic pathways, we conducted decuple-genes regulation to optimize metabolic flux through the 1-deoxy-d-xylulose-5-phosphate (DXP) biosynthesis pathway for overproduction of the isoprenoid, lycopene, in a lycopene degradation pathway-blocked *C. glutamicum* strain Δ*crtY*_*e/f*_Δ*crtEb*. Ten endogenous genes (*dxs*, *dxr*, *ispD*, *ispE*, *ispF*, *ispG*, *ispH*, *crtE*, *crtB*, and *crtI*) in lycopene biosynthesis pathway were targeted for metabolic reprogramming (Fig. 3a). Because some DXP pathway genes are essential genes, it is impossible to knock out all the ten original copies or even replace their RBSs with the tailored GGGGGGGG RBS due to its low translation efficiency (data not shown). To rapidly insert the tailored GGGGGGGG RBS upstream target genes, the ten genes were organized into three artificial clusters and integrated into *C. glutamicum* chromosome. The artificial *dxs*-*dxr*-*ispD* and *ispE*-*ispF*-*ispG*-*ispH* clusters with tailored GGGGGGGG RBSs were inserted at the CGP2 prophage region and *upp* gene, respectively, as a second copy. The artificial *crtE*-*crtB*-*crtI* cluster with tailored GGGGGGGG RBSs was inserted between *cgl0622* and *cgl0627* to replace the native cluster (Fig. 3a). The engineered ancestral strain produced 0.61 mg/gCDW lycopene. With the increase of target gene number, the discreteness of genetic combinations generated by BETTER became more important for producing a high-quality library. The base editing window can be adjusted using truncated or extended gRNAs^27^. Therefore, we tested two sets of double-gRNAs combinations, 20 + 20-nt gRNAs, and 20 + 18-nt gRNAs (Fig. 3b). The 20 + 18-nt gRNAs led to more moderate editing of the second to fourth Gs than the 20 + 20-nt gRNAs (Fig. 3c and Supplementary Data 4). We then merged the RBS variants generated by BETTER with the two sets of gRNAs and observed large decreases in KL divergence values for all the ten edited RBSs, suggesting a more even distribution of RBS variants for each target gene (Fig. 3d, e and Supplementary Data 4).

Targeted sequencing revealed the G/A/C/T-containing RBS types were 3,550 for *dxs* (including 237 G/A-containing types), 1,873 for *dxr* (including 221 G/A-containing types), 1,847 for *ispD* (including 238 G/A-containing types), 3,061 for *ispE* (including 237 G/A-containing types), 1,694 for *ispF* (including 237 G/A-containing types), 1,666 for *ispG* (including 223 G/A-containing types), 1,987 for *ispH* (including 236 G/A-containing types), 3,386 for *crtE* (including 245 G/A-containing types), 2,263 for *crtB* (including 236 G/A-containing types), and 1,667 for *crtI* (including 231 G/A-containing types) (Supplementary Data 4). It was estimated that the theoretically maximum library complexity could reach 2.7×10^33^ if a large enough population of cells were examined. Screening of lycopene overproducing variants was done by isolating colonies with intense red pigmentation on CGXII minimal medium supplemented with glucose (Fig. 3f). Variants were isolated from approximately 1 × 10^5^ colonies and some produced as much as 4.8-fold lycopene relative to the ancestral strain (Fig. 3g). Under similar experimental conditions, our highest lycopene yield of 2.9 mg/gCDW is better than the documented record for lycopene production by *C. glutamicum* from glucose (2.4 mg/gCDW)^28^.

Targeted sequencing revealed the rich diversity of RBSs in the four overproducing variants. Although no consistent expression pattern for the ten lycopene biosynthetic genes was identified in current experiment, we noticed that *crtI* catalyzing the last step of lycopene biosynthesis was controlled by a strong RBS (GAAAGGGG) in all the four overproducers (Fig. 3g and Supplementary Data 5). This suggests that the conversion of phytoene to lycopene might be a rate-limiting step. Since the proportion of GAAAGGGG ranked sixth in the RBS_*crtI*_ library generated by BETTER and exhibited the highest translation strength among the six RBSs with the largest proportion, it was easily enriched when the high-level expression of *crtI* benefited lycopene biosynthesis (Supplementary Fig. 8). Interestingly, in three out of four overproducing variants (strains 2–4), the second copy of *dxr* and *ispD* were missing from the artificial cluster on the chromosome, which may be induced by the multisite single-stranded cleavages by nCas9. And the fourth overproducing variant (strain 1) had a very weak RBS for *dxr* (Fig. 3g and Supplementary Data 5). Therefore, we reasoned that too much expression of *dxr* and *ispD* might reduce lycopene production and their original copies on *C. glutamicum* chromosome are sufficient. We then overexpressed *dxr* and *ispD* via a plasmid in strains 2 and 4 and observed the expected decrease in lycopene titer (Supplementary Fig. 9), which validated our hypothesis. For a biosynthetic pathway, proteins expressed at suboptimal levels could lead to problems, such as protein burden, accumulation of toxic intermediates, or metabolic imbalance^29^. It is speculated that overexpression of *dxr* and *ispD* might overburden the host and negatively affect lycopene biosynthesis in this case. To verify the improvement in lycopene biosynthesis is solely due to RBS editing, we rebuilt strains 2 and 4 from the wild-type strain. Three rounds of homologous recombination were conducted to insert three artificial clusters with the edited RBS combinations. The rebuilt strain 2 showed almost the same lycopene production capability as the screened strain 2, while the rebuilt strain 4 produced slightly more lycopene than the screened strain 4 (Supplementary Fig. 10). The results demonstrate that the RBS engineering dominantly leads to the phenotype change. The optimized DXP biosynthesis pathway holds the potential to produce other useful isoprenoids^30^.

### Application of BETTER in diversifying 5′ UTR and promoter

**Fig. 4:**
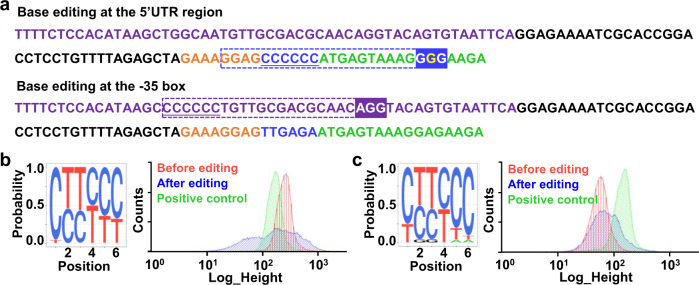
Diversifying 5′ UTR and promoter by BETTER for regulating gene expression. **a** Design of base editing targets by replacing the original 5′ UTR between RBS and start codon and −35 box of the promoter to six consecutive Cs, respectively. The target Cs are underlined. The N20 sequences of gRNA are denoted with the dotted box. PAMs for 5′ UTR and promoter editing are shaded in blue and purple, respectively. The mutated G for the generation of a GGG PAM is highlighted in yellow. The original sequence for *gfp* expression cassette is shown in Supplementary Fig. 1. **b**, **c** Analyses of the 5′ UTR (**b**) and −35 box (**c**) libraries by NGS and flow cytometry. Three colonies were used for base editing and the cells were mixed with an equal proportion before extraction of genomic DNAs, PCR amplification, and NGS. The original *gfp* expression cassette was used as a positive control.

Besides RBS, the promoter and the 5′ UTR between RBS and the translational start codon also play important roles in controlling gene expression^25,26^. To expand the application of BETTER in diversifying other translation or transcription elements, we tested library construction for the 5′ UTR between RBS and the start codon and the −35 box of the promoter. The chromosomal *gfp* expression cassette in *C. glutamicum* was modified to change the original 5′ UTR sequence from TTGAGA to six consecutive Cs, which could be edited by base editors to generate a C/T-rich library (Fig. 4a). Construction of a G/A-rich library initiated from consecutive Gs was not selected here to avoid its interference with the upstream RBS. Because there is no available NGG PAM downstream the target Cs, we introduced a synonymous mutation (GGA to GGG) to generate a GGG PAM. Because the target Cs do not locate at the editing window of Target-AID base editing tool (positions −20 to −16, counting the PAM as positions 1–3)^13^, we used BE3^12^ base editing tool with a larger editing window in *C. glutamicum* (positions −19 to −11)^31^ to cover the target six Cs in 5′ UTR (Fig. 4a). After base editing, a C/T-rich 5′ UTR library was generated. Because a uracil DNA glycosylase inhibitor was included in BE3, few C to G and C to A conversions were observed (Fig. 4b). Although the C distal to PAM was less efficiently edited, all the 64 C/T-containing combinations were detected by NGS (Supplementary Data 6). The discreteness of 5′ UTR variants generated by BETTER was evaluated using the KL divergence analysis and a relatively low value of 1.98 was obtained (Supplementary Fig. 11). Totally, 772 out of 4,096 kinds of G/A/C/T-containing combinations were detected (Supplementary Data 6). By analyzing GFP expression using flow cytometry, it was found that the edited cells showed diverse GFP fluorescence signals (Fig. 4b).

To regulate gene expression by editing promoter, the original −35 box of promoter *P*_11F_ used for *gfp* expression on *C. glutamicum* chromosome was changed from TGGCAA to six consecutive Cs. An available AGG PAM downstream the target Cs allowed direct BETTER application using Target-AID base editing tool (Fig. 4a). Base editing targeting the six Cs also generated all the 64 C/T-containing combinations with a KL divergence value of 1.95 (Supplementary Fig. 11). Totally, 900 G/A/C/T-containing combinations were detected (Supplementary Data 6). The GFP fluorescence outputs of the edited cell cultures were diversified as expected (Fig. 4c). The results suggest that the BETTER method can also be used to diversify gene expression by generating libraries for other translation or transcription elements than RBS, such as 5′ UTR and promoter.

### Application of BETTER in *B. subtilis*

**Fig. 5:**
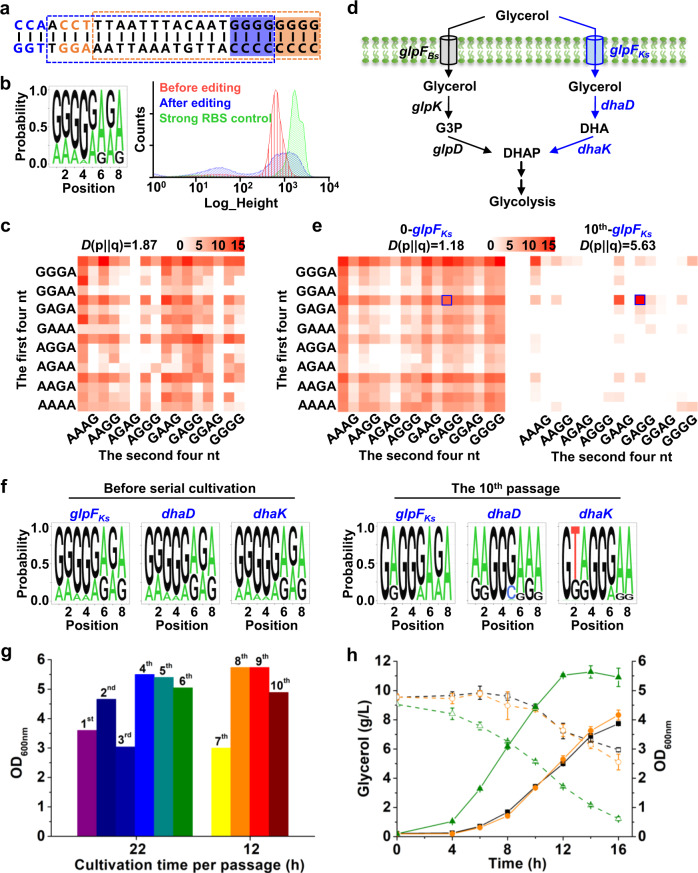
Regulating *gfp* expression and reprogramming glycerol catabolism by BETTER in *B. subtilis*. **a** Two interlaced gRNAs facilitate editing of eight Gs. A set of PAM, gRNA, and editing window is shown in the same color. **b** Analyses of the RBS_*gfp*_ library by NGS and flow cytometry. Three colonies were used for base editing and the cells were mixed with an equal proportion before extraction of genomic DNAs, PCR amplification, and NGS. A strong RBS (AGGAGGCG)^39^ was used as a control. **c** The matrix shows the coverage and discreteness of 256 G/A-containing RBS variants in the RBS_*gfp*_ library generated by BETTER with double gRNAs. Scale bar represents log_2_(relative proportion). KL divergence was used to evaluate the discreteness of genetic combinations. **d** The native glycerol catabolism pathway of *B. subtilis* (black arrows) and the heterologous pathway from *Klebsiella* sp. (blue arrows). G3P glyceraldehyde 3-phosphate, DHA dihydroxyacetone, DHAP dihydroxyacetone phosphate, *glpF*_*Bs*_ glycerol facilitator from *B. subtilis*; *glpK* glycerol kinase, *glpD* G3P dehydrogenase, *glpF*_*Ks*_ glycerol facilitator from *Klebsiella* sp., *dhaD* glycerol dehydrogenase, *dhaK* dihydroxyacetone kinase. **e** The matrixes show the coverage and discreteness of 256 G/A-containing RBS variants in the RBS libraries of *glpF*_*Ks*_ generated by BETTER before (0) and after ten passages (tenth) of serial cultivation in glycerol. Scale bar represents log_2_(relative proportion). Blue squares represent the enriched RBS_*glpFKs*_ GAGGGAGA. The matrixes for RBS libraries of *dhaD* and *dhaK* are shown in Supplementary Fig. 14. Three colonies were used for base editing and the cells were mixed with an equal proportion before extraction of genomic DNAs, PCR amplification, and NGS. **f** Components of RBS libraries before and after ten passages of serial cultivation in glycerol. **g** Serial cultivation for screening genetic combinations that favor growth on glycerol. The numbers above columns represent the passages of serial cultivation. For the first six passages, transfer was made after 22 h cultivation. For the subsequent four passages, the cultivation time for each passage was reduced to 12 h because the cell growth rate largely increased. **h** Growth (filled symbols) and glycerol consumption (open symbols) of wild-type *B. subtilis* 168 (black square), the starting strain (orange circle) with heterologous *glpF*_*Ks*_, *dhaD*, and *dhaK* genes controlled by GGGGGGGG RBSs, and the screened strain (green triangle) from the tenth passage with RBS_*glpFKs*_ GAGGGAGA, RBS_*dhaD*_ AAGGGAAA, and RBS_*dhaK*_ GTAGGGAA. Values and error bars reflect the mean ± s.d. of three biological replicates (*n* = 3). Source data underlying Fig. 5c, e, g, h are provided as a Source Data file.

In order to further verify the general applicability of the BETTER method for microorganisms other than *C. glutamicum*, the method was expanded to another model bacterium *B. subtilis*^32^, in which we have recently developed an efficient base editing technology^33^. *B. subtilis* is widely used for the bioproduction of antibiotics, vitamins, and proteins, whereas its genome engineering is more difficult than genetically tractable hosts such as *E. coli*^33^. GFP was first used as a reporter to test the BETTER method. An expression cassette consisting of a constitutive promoter, the tailored GGGGGGGG RBS, and *gfp* was integrated into the *B. subtilis* chromosome (Supplementary Fig. 12). A previously constructed Target-AID base editing tool plasmid^33^ expressing a catalytically dead Cas9 (dCas9)–cytidine deaminase fusion and two interlaced RBS-targeting gRNA was used for RBS library construction (Fig. 5a). NGS analysis suggests all the eight Gs were edited. The strength of the generated G/A-rich RBS library spanned nearly three orders of magnitude in terms of GFP fluorescence intensity (Fig. 5b), demonstrating the good performance of BETTER in *B. subtilis*. A detailed analysis reveals the generation of 230 out of 256 G/A-containing RBS variants with a KL divergence value of 1.87 (Fig. 5c), and the number increases to 3,519 in the case of calculating G/A/C/T-containing RBS variants (Supplementary Data 7).

After proving the applicability of BETTER in *B. subtilis*, we reprogramed the glycerol catabolism pathway to accelerate glycerol utilization in *B. subtilis*. Glycerol is a major by-product of biodiesel production and a promising substrate for biomanufacturing^34^. Notably, glycerol is not only a natural carbon source for *B. subtilis* but also enhances the bioproduction of some valuable chemicals (e.g., menaquinone-7 and uridine) when used as the feedstock for *B. subtilis*^35,36^. A heterologous glycerol catabolism pathway from an efficient glycerol utilizer *Klebsiella* sp. M5a1 (ATCC BAA-1236)^37^ was introduced into *B. subtilis* 168 (Fig. 5d). The indigenous glycerol catabolism pathway was not selected for optimization to bypass the regulation by anti-terminator protein GlpP^38^. An artificial cluster consisting of a constitutive promoter *P*_*gapDH*_^39^, *glpF*_*Ks*_ (encoding glycerol facilitator), *dhaD* (encoding glycerol dehydrogenase), and *dhaK* (encoding dihydroxyacetone kinase) from *Klebsiella* sp. was integrated into the *B. subtilis* chromosome. The tailored GGGGGGGG RBS was installed upstream of each target gene (Supplementary Fig. 13). Then, BETTER was used to generate RBS libraries for three heterologous genes. NGS analysis of the edited cells suggests successful RBS diversification for three targets. Nearly all the 256 G/A-containing RBS variants were detected, 254 types for RBS_*glpFKs*_, 254 types for RBS_*dhaD*_, and 255 types for RBS_*dhaK*_ (Fig. 5e, f and Supplementary Data 8). In the case of calculating the G/A/C/T-containing RBS variants, the types of RBS_*glpFKs*_, RBS_*dhaD*_, and RBS_*dhaK*_ variants were 7,170, 3,909, and 4,913, making the estimated theoretically maximum library complexity of 1 × 10^11^ (7,170 × 3,909 × 4,913) (Supplementary Data 8).

After curing of the tool plasmid, the edited cell mixture was serially cultivated in M9 minimal medium with glycerol as a sole carbon source to enrich variants with improved glycerol utilization capability. For the first passage, it took 22 h for the culture to reach the highest biomass. After six passages of serial cultivation, the cultivation time was reduced to 12 h, suggesting an improvement of cell growth rate and an enrichment of better glycerol utilizing strains (Fig. 5g). NGS analysis of the cells before and after ten passages of serial cultivation shows that RBS_*glpFKs*_ GAGGGAGA, RBS_*dhaD*_ AAGGGAAA, and RBS_*dhaK*_ GTAGGGAA were enriched in varying degrees (Fig. 5f and Supplementary Fig. 14). Two fast-growing single colonies picked from the tenth passage had the same aforementioned RBS combinations. We performed fermentation of one screened strain in shake flask to determine its improved glycerol utilization capability using the wild-type strain and the starting strain without base editing as controls. The growth rate and glycerol utilization rate of the screened strain were apparently higher than those of the controls (Fig. 5h). The improvement demonstrates the applicability of BETTER in metabolic engineering of *B. subtilis*.

## Discussion

Here we demonstrate that base editors can be used to generate localized diversity within the expression regulatory components of target genes and that the combinatorial library can be selected or screened to explore the metabolic potential of microorganisms. This method, BETTER, can simultaneously target up to ten genes in *C. glutamicum* and *B. subtilis* and diversify their expression levels by just one round of base editing. To the best of our knowledge, it is the highest number of simultaneously regulated genes in any microorganisms besides *E. coli*, which can be engineered via the MAGE method for 24 genetic components with 35 MAGE cycles^6^. CRISPR-guided cytidine deaminase has been demonstrated for simultaneous editing of up to 41 chromosomal targets in bacteria^19^, suggesting great potential of BETTER in regulating large numbers of target genes for reprogramming complex metabolism. The simple base editing mechanism could make this method amenable for use in other eukaryotic or prokaryotic microorganisms with low efficiency of transformation and recombination or low tolerance to DSBs and Cas9 toxicity, such as *Yarrowia lipolytica*^40^, *Aspergillus niger*^41^, streptomycetes^42^, and clostridia^43^ that are reported to be compatible with base editing techniques.

Using BETTER, we highlight several samples of gene expression regulation for metabolic reprogramming. First, we show that libraries of RBS, 5′ UTR, and promoter can be easily generated by BETTER in situ and span several orders of magnitude of protein expression. In addition, an optimized expression pattern for xylose catabolic genes was demonstrated, suggesting that high-level expression of xylose catabolic genes, especially *xylA* and *tkt*, may benefit xylose uptake. However, it is a different case for reprogramming lycopene biosynthesis. Too much expression of two lycopene biosynthetic genes, *dxr* and *ispD*, would reduce lycopene production. Additionally, we observed that more than one expression pattern for the ten target genes can lead to lycopene overproduction, which is probably different local maximums in the whole production optimization landscape. These insights may inform the study of lycopene biosynthesis regulation and future design of lycopene microbial cell factories.

BETTER presents a number of significant advantages for diversifying multigene expression. Compared to previous methods, BETTER does not require synthesis, transformation, or incorporation of heterologous DNA donors and avoids the introduction of lethal DSBs. Considering the high editing efficiency and over 90% cell survival rate of base editing techniques^18^, considerable genetic combinations could be generated for regulating a large number of target genes by just one cycle of base editing. BETTER might be particularly important for engineering industrial strains, which are known to be notoriously hard to perform sophisticated genetic manipulations. Admittedly, a tailored translation or transcription element must be inserted upstream to each target prior to base editing. With the cost and time of DNA synthesis and sequencing rapidly decreasing^44,45^, de novo synthesis of an artificial cluster preloaded with tailored translation or transcription elements will make this preparation step cost- and time-efficient. Codon optimization can also be conducted for heterologous genes during DNA synthesis to overcome the potentially negative effects of rare codons on gene expression. The advent of CRISPR/Cas-based gene-editing techniques allows knock-in of gene clusters even in genetically intractable microorganisms^46^, which might enable BETTER in a much wider range of hosts. Currently, base editors with different base transition capabilities, broad PAM compatibility, and reduced off-target activity are being created^14,47–50^, which will promote the future evolution of BETTER. For instance, another version of BETTER based on ABEs^14^ with higher product purity (typically ≥ 99.9%) than CBEs can be used to generate a rigorous G/A-containing RBS library starting from a tailored RBS with consecutive As. Taken together, we envision that BETTER will greatly improve our capability in terms of sophisticated metabolic reprogramming in microorganisms beyond common laboratory strains like *E. coli* and *S. cerevisiae*.

## Methods

### Strains and cultural conditions

Strains used in this study are listed in Supplementary Data 9. *E. coli* strain DH5α that was used for general cloning was cultivated aerobically at 37 °C in Luria–Bertani (LB) broth. Kanamycin (Km, 50 μg/mL) or chloramphenicol (Cm, 20 μg/mL) was added to the medium as required. *C. glutamicum* strain ATCC 13032 and its derivatives were cultivated aerobically at 30 °C in LB broth supplemented with 10 g/L glucose (LBG medium) or CGXII minimal medium^18^ supplemented with appropriate glucose or xylose. Km (25 μg/mL) or Cm (5 μg/mL) was added as required. *B. subtilis* strain 168 and its derivatives were cultivated aerobically at 37 °C in LB broth or M9 minimal medium supplemented with appropriate glycerol. Cm (5 μg/mL) was added as required.

### Plasmid construction

Plasmids used in this study are listed in Supplementary Data 9. Primers used for plasmid construction and the construction process are included in Supplementary Data 10. Plasmids were constructed via recombination or Golden Gate assembly. Recombination was conducted using the ClonExpress MultiS One Step Cloning Kit (Vazyme, Nanjing, China). Golden Gate assembly protocols were designed using j5 software tool (Revision as of 10:05, 18 October 2018 by J5admin)^51^.

### Design of gene expression cassette

The gene expression cassettes for BETTER application in *C. glutamicum* and *B. subtilis* are shown in Supplementary Fig. 1 and Supplementary Fig. 12, respectively. Constitutive promoters *P*_*11F*_ and *P*_*gapDH*_ were used for gene transcription in *C. glutamicum* and *B. subtilis*, respectively^39,52^. The tailored GGGGGGGG RBS was inserted upstream of the target gene. To determine the location of introducing the tailored GGGGGGGG RBS relative to the location of a start codon, it is suggested to follow the rules concluded from the transcriptome analysis of the target host. For example, an analysis of *C. glutamicum* transcriptome suggests that the spacers between RBS and translational start codon are dominantly 5–10 nucleotides^53^. Therefore, a spacer of six nucleotides (TTGAGA) that has been experimentally verified for gene expression in *C. glutamicum* was inserted between the RBS and the start codon in this study. Such transcriptome analyses are usually available for a model or extensively studied microorganisms. However, for some non-model microorganisms without such analyses, it is suggested to use experimentally verified RBS and spacer sequences and change the RBS to consecutive Gs for the generation of RBS diversity using the BETTER method.

### Chromosomal integration

A pK18*mobsacB* derived plasmid that harbors two ~1000 bp homologous arms flanking at both sides of the target fragment was used to integrate heterologous genes and tailored translation or transcription elements into *C. glutamicum* chromosome^54^. In situ replacement of the native *crtE*, *crtB*, and *crtI* cluster with an artificial cluster preloaded with tailored RBSs was achieved using the CRISPR/Cas9-based gene replacement method^52^. Specifically, pgRNA-*crtEBI* plasmid harboring two gRNA cassettes targeting the two ends of the native cluster (N20: 5′-AAGCGCCATATACAACGATT-3′; N20: 5′-ACAGAAAGAATTCGTGATGA-3′) was co-transformed into *C. glutamicum* with a pCas9-*crtEBI* plasmid expressing Cas9 and carrying the homologous arms and artificial *crtEBI* cluster preloaded with tailored GGGGGGGG RBSs via electroporation. Cells were spread on SGY plates^52^ supplemented with Km, Cm, and isopropyl-β-d-thiogalactopyranoside (IPTG) (0.01 mM) immediately after recovery. After 2–3 days of cultivation, colonies were verified by PCR. Plasmids were cured of edited cells by cultivation at 37 °C in antibiotic-free LBG medium.

Gene integration in *B. subtilis* was performed using the CRISPR/Cas9-based genome editing method^55^. pBAC-Cas9-Δ*amyE* derived plasmids (Supplementary Data 9) that express Cas9 and gRNA targeting *amyE* and carry homologous arms flanking at both sides of the target fragment were used to integrate heterologous genes and tailored RBSs into the *amyE* locus of *B. subtilis* chromosome. After gene integration, the tool plasmid was cured of edited cells by the serial culture at 37 °C in antibiotic-free LB medium^56^.

### Generation of genetic variants with base editing

The pnCas9(D10A)-AID^Ts^ plasmid expressing nCas9(D10A)-AID was co-transformed into *C. glutamicum* with a pgRNA plasmid expressing one or two gRNAs targeting the tailored translation or transcription elements via electroporation. Three PCR-verified transformants were cultivated in LBG medium supplemented with Km, Cm, and IPTG (1 mM) for 12 h. Base editing using the BE3 base editor was conducted following the same procedure but using the pXMJ19-rACU^TS^ tool plasmid. The pBAC-dCas9-AID-gRNA-RBS2^TS^ plasmid expressing Target-AID base editor and two gRNAs targeting the tailored GGGGGGGG RBSs was transformed into *B. subtilis* for base editing. Three PCR-verified transformants were cultivated in LB medium supplemented with Cm and IPTG (0.1 mM) for 12 h^33^.

### NGS and data analysis

Base-edited cell cultures of three biological replicates were mixed with an equal proportion. Approximately, 10^9^ cells were used for extraction of genomic DNAs. DNA fragments containing the target region (200–250 bp) were amplified from the extracted genomic DNAs by PCR using primers listed in Supplementary Data 10. NEBNext Ultra DNA Library Prep Kit was used to convert the amplicon into indexed libraries for NGS on the Illumina platform. Library construction and sequencing were performed by GENEWIZ (Suzhou, China). Approximately 100,000 reads per sample were analyzed. For NGS data analysis, the 20-nt sequence upstream of the target region was used to locate the position of the target region in each read via the blast. The sequence of the target region was then extracted from the reading and mapped to the reference sequence to analyze the base editing event. Finally, the number of each RBS variant was counted using the R package (v4.0.2). The sequence logo was generated using the statistical result of G/A/C/T-containing RBS variants and the R package ‘ggseqlogo’ (v0.1). The heat map was generated using the statistical result of G/A-containing RBS variants and the R package ‘ggplot2’ (v3.3.2).

### Flow cytometry

Cells were harvested from cultures by centrifugation at 6,000×*g* for 10 min, washed once, and resuspended in phosphate buffer (pH 7.4). GFP fluorescence was analyzed by flow cytometry (FACS, Beckman Coulter MoFlo XDP) with the following parameters: excitation at 488 nm, emission fluorescence at 529 ± 14 nm, sample pressure of 60 psi. The nozzle diameter was 70 μm. Cells were captured on the signal channels of FITC (voltage 510 V), FSC (voltage 150 V), and SSC (voltage 250 V). All captured events were used for fluorescence analysis. Data were analyzed using the Beckman Summit software v5.2.

### Serial cultivation and assay for xylose utilization

Base-edited *C. glutamicum* cultures were cultivated in the antibiotic-free medium at 37 °C to cure tool plasmids. Serial cultivation was performed at 30 °C and with shaking at 220 rpm using CGXII minimal medium supplemented with 10 g/L xylose. After cultivated for 24 h, the culture was used as a seed to inoculate fresh medium with an initial optical density at 600 nm (OD_600 nm_) of 0.1, which was then incubated under the same conditions. For each passage, cells were collected for genomic DNA extraction, which was used for NGS of RBS regions. After six passages, the culture was diluted, plated on CGXII agar plates supplemented with 10 g/L xylose, and incubated at 30 °C. The colonies that grew fast were picked and analyzed for RBSs of *xylA*, *xylB*, and *tkt*. To assay xylose utilization capability, screened strains were inoculated into 250 mL shake flasks with 50 mL CGXII minimal medium supplemented with 10 g/L xylose. Samples were picked periodically and analyzed for OD_600 nm_ and residual xylose using high-performance liquid chromatography (HPLC)^57^.

### Colorimetric screening and assay for lycopene production

Base-edited and plasmid-cured cell cultures were plated on CGXII agar plates supplemented with 18 g/L glucose (100 mM) and incubated at 30 °C for 72 h to produce red colonies. In total, 96 colonies with increased color intensity by visual inspection were picked and cultivated in 96-well plates with 600 μL CGXII minimal medium supplemented with 18 g/L glucose. Cells at the exponential growth phase were transferred into 96-well plates with fresh medium for lycopene production. After 24 h cultivation, cells were harvested by centrifugation at 6,000×*g* for 10 min for a second round of screening by visual inspection. Several strains with relatively high color intensity were tested for lycopene production in 250 mL shake flasks with 50 mL fresh medium. Lycopene was extracted with methanol:acetone mixture (7:3) at 60 °C for 80 min with thorough vortexing every 20 min and quantified with HPLC^28^.

### Serial cultivation and assay for glycerol utilization

Base-edited *B. subtilis* cultures were cultivated in the antibiotic-free medium at 37 °C to cure the tool plasmid. Serial cultivation was performed at 37 °C and with shaking at 220 rpm using M9 minimal medium supplemented with 10 g/L glycerol. After cultivated for 12–22 h, the culture was used as a seed to inoculate fresh medium with an initial OD_600 nm_ of 0.1, which was then incubated under the same conditions. Cells were collected before the serial cultivation and from the tenth passage for genomic DNA extraction, which was used for sequencing analysis of RBS regions. After ten passages, the culture was diluted, plated on M9 agar plates supplemented with 10 g/L glycerol, and incubated at 37 °C. The colonies that grew fast were picked and analyzed for RBSs of *glpF*_*Ks*_, *dhaD*, and *dhaK*. To assay glycerol utilization capability, screened and control strains were inoculated into 250 mL shake flasks with 50 mL M9 minimal medium supplemented with 10 g/L glycerol. Samples were picked periodically and analyzed for OD_600 nm_ and residual glycerol using HPLC^34^.

### Assay for RBS strength

*C. glutamicum* strain harboring a chromosomal *gfp* expression cassette was used for analyzing the strength of RBS variants. CRISPR/Cas9-mediated ssDNA recombineering^52^ was applied to replace the original GGGGGGGG RBS of *gfp* with different RBSs. Specifically, a pgRNA-*recT*-RBS plasmid harboring a *recT* expression cassette and a gRNA cassette targeting the original GGGGGGGG RBS (N20, 5′-CCCCCCCCTAGCTCTAAAAC-3′) was first transformed into the *gfp* expressing *C. glutamicum*. The transformant was then co-transformed with a pCas9 plasmid expressing Cas9 and 90mer synthetic ssDNAs containing an RBS variant (Supplementary Data 10). Cells were spread on SGY plates supplemented with Km, Cm, and IPTG (0.01 mM) immediately after recovery. After 2–3 days of cultivation, colonies were verified by PCR and sequencing. Plasmids were cured of edited cells by cultivation at 37 °C in antibiotic-free LBG medium. Edited cells were cultivated, harvested by centrifugation at 6,000×*g* for 10 min, washed once, and re-suspended in phosphate buffer (pH 7.4). GFP fluorescence intensity was determined using a microplate reader (SpectraMax M5, Molecular Devices, *λ* excitation = 488 nm, *λ* emission = 520 nm).

### Enzyme activity assay

*C. glutamicum* cells at exponential phase were harvested and washed twice with 50 mM potassium phosphate buffer (pH 7.4). The cell pellet was resuspended in the same buffer and disrupted by bead beating using a mechanical disruptor (FastPrep^®^24, MP). The lysed cells were centrifuged at 18,000×*g* for 30 min at 4 °C, and the supernatant was used for activity assay. Xylose isomerase (XylA) activity was assayed with a reaction mixture containing 100 mM Tris-HCl buffer (pH 7.5), 10 mM MgCl_2_, 0.15 mM NADH, 500 mM xylose, and 2 U sorbitol dehydrogenase (Sigma, USA). NADH consumption was measured spectrophotometrically at 340 nm at 25 °C. One unit of xylose isomerase activity was defined as the amount of enzyme that consumes 1 μmol NADH per minute. Xylulokinase (XylB) activity was measured with a reaction mixture contained 100 mM Tris-HCl buffer (pH 7.8), 5 mM MgSO_4_, 0.2 mM NADH, 5 mM d-xylulose, 1 mM ATP, 1.5 mM phosphoenolpyruvate, 3 U pyruvate kinase (Sigma, USA), and 3 U l-lactate dehydrogenase (Sigma, USA). NADH consumption was measured spectrophotometrically at 340 nm at 25 °C. One unit of xylulokinase activity was defined as the amount of enzyme that consumes 1 μmol NADH per minute. Transketolase (Tkt) activity was measured with a reaction mixture containing 50 mM glycylglycine buffer (pH 8.5), 0.3 mM thiamine pyrophosphate, 1 mM xylulose-5-phosphate, 1 mM ribose-5-phosphate, 0.2 mM NADH, 1 U glycerophosphate dehydrogenase (Sigma, USA), and 10 U purified triose phosphate isomerase. NADH consumption was measured spectrophotometrically at 340 nm at 25 °C. One unit of transketolase activity was defined as the amount of enzyme that consumes 1 μmol NADH per minute.

### Statistics

Values and error bars reflect the mean ± s.d. of three biological replicates (*n* = 3). All *P* values were generated from a two-sided *t* test (*n* = 3) using Microsoft Excel 2016 (Microsoft Corporation). KL divergence was calculated using Eq. 1 and Microsoft Excel 2016 (Microsoft Corporation).

1 $$D\left( {p||q} \right) =\sum_{i = 1}^n {p\left( x \right)\log } \frac{{p(x)}}{{q(x)}}$$

### Reporting summary

Further information on research design is available in the Nature Research Reporting Summary linked to this article.

## Supplementary information

Supplementary Information

Peer Review File

Reporting Summary

Description of Additional Supplementary Files

Supplementary Data 1

Supplementary Data 2

Supplementary Data 3

Supplementary Data 4

Supplementary Data 5

Supplementary Data 6

Supplementary Data 7

Supplementary Data 8

Supplementary Data 9

Supplementary Data 10

## Data Availability

The data supporting the findings of this work are available within the paper and the Supplementary Information files. A reporting summary for this article is available as a Supplementary Information file. The data sets generated and analyzed during this study are available from the corresponding author upon request. The raw reads of the NGS data were deposited into the Sequence Read Archive database of NCBI under accession number PRJNA608771. Source data are provided with this paper.

## Source data

Source Data
