# Supplementary material for: In-situ generation of large numbers of genetic combinations for metabolic reprogramming via CRISPR-guided base editing
Source: Nat Commun. 2021 Jan 29;12:678. doi: 10.1038/s41467-021-21003-y (PMC7846839; doi:10.1038/s41467-021-21003-y)
Supplement: Supplementary file 1 — Supplementary Information [file 41467_2021_21003_MOESM1_ESM.pdf]

In-situ generation of large numbers of genetic combinations for metabolic reprogramming via CRISPR-guided base editing

Wang *et al.*

a

TTTTCTCCACATAAGCTGGCAATGTTGCGACGCAACAGGTACAGTGTAATTCAGGAGAAAATCGCACCGGA
CCTCCTGTTTTAGAGCTAGAAAGGAGTTGAGAATGAGTAAAGGAGAAGAACTTTTCACTGGAGTTGTCCCA
ATTCTTGTTGAATTAGATGGTGATGTTAATGGGCACAAATTTTCTGTCAGTGGAGAGGGTGAAGGTGATGC
AACATACGGAAAACTTACCCTTAAATTTATTTGCACTACTGGAAAACTACCTGTTCCATGGCCAACACTTGT
CACTACTTTCGCGTATGGTCTTCAATGCTTTGCGAGATACCCAGATCATATGAAACAGCATGACTTTTTCAA
GAGTGCCATGCCCGAAGGTTATGTACAGGAAAGAACTATATTTTTCAAAGATGACGGGAACTACAAGACA
CGTGCTGAAGTCAAGTTTGAAGGTGATACCCTTGTTAATAGAATCGAGTTAAAAGGTATTGATTTTAAAGAA
GATGGAAACATTCTTGGACACAAATTGGAATACAACTATAACTCACACAATGTATACATCATGGCAGACAA
ACAAAAGAATGGAATCAAAGTTAACTTCAAAATTAGACACAACATTGAAGATGGAAGCGTTCAACTAGCAG
ACCATTATCAACAAAATACTCCAATTGGCGATGGCCCTGTCCTTTTACCAGACAACCATTACCTGTCCACA
CAATCTGCCCTTTCGAAAGATCCCAACGAAAAGAGAGACCACATGGTCCTTCTTGAGTTTGTAACAGCTGC
TGGGATTACACATGGCATGGATGAACTATACAAATAA

b

TTTTCTCCACATAAGCTGGCAATGTTGCGACGCAACAGGTACAGTGTAATTCAGGAGAAAATCGCACCGGA
CCTCCTGTTTTAGAGCTAGGGGGGGGTTGAGAATGAGTAAAGGAGAAGAACTTTTCACTGGAGTTGTCCCA
ATTCTTGTTGAATTAGATGGTGATGTTAATGGGCACAAATTTTCTGTCAGTGGAGAGGGTGAAGGTGATGC
AACATACGGAAAACTTACCCTTAAATTTATTTGCACTACTGGAAAACTACCTGTTCCATGGCCAACACTTGT
CACTACTTTCGCGTATGGTCTTCAATGCTTTGCGAGATACCCAGATCATATGAAACAGCATGACTTTTTCAA
GAGTGCCATGCCCGAAGGTTATGTACAGGAAAGAACTATATTTTTCAAAGATGACGGGAACTACAAGACA
CGTGCTGAAGTCAAGTTTGAAGGTGATACCCTTGTTAATAGAATCGAGTTAAAAGGTATTGATTTTAAAGAA
GATGGAAACATTCTTGGACACAAATTGGAATACAACTATAACTCACACAATGTATACATCATGGCAGACAA
ACAAAAGAATGGAATCAAAGTTAACTTCAAAATTAGACACAACATTGAAGATGGAAGCGTTCAACTAGCAG
ACCATTATCAACAAAATACTCCAATTGGCGATGGCCCTGTCCTTTTACCAGACAACCATTACCTGTCCACA
CAATCTGCCCTTTCGAAAGATCCCAACGAAAAGAGAGACCACATGGTCCTTCTTGAGTTTGTAACAGCTGC
TGGGATTACACATGGCATGGATGAACTATACAAATAA

Supplementary Fig. 1. Sequence of the *gfp* expression cassette used in *C. glutamicum*. a, The *gfp* expression cassette used as the strong RBS control in Fig. 1d. **b,** The *gfp* expression cassette with the RBS changed from GAAAGGAG to the tailored GGGGGGGG for editing. Purple fonts, P_{11F} promoter; orange fonts, RBS; blue fonts, 5'UTR between RBS and *gfp*; green fonts, *gfp*.

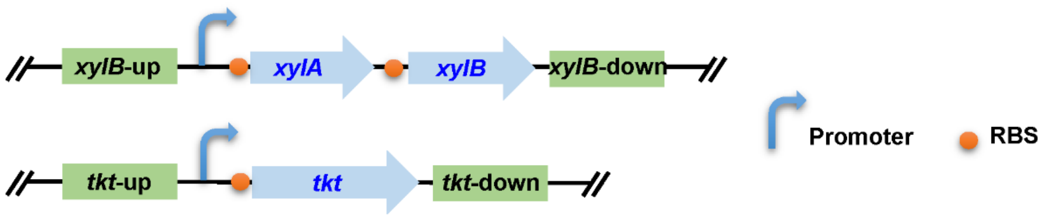

Supplementary Fig. 2. Schematic illustration of integration of heterogeneous *xylA*, promoter and tailored GGGGGGGG RBS into *C. glutamicum* chromosome. A constitutive promoter, a tailored GGGGGGGG RBS, *xylA* gene from *E. coli*, and a second tailored GGGGGGGG RBS were first integrated into the upstream of *xylB* gene. The same constitutive promoter and tailored GGGGGGGG RBS were then integrated into the upstream of *tkt* gene.

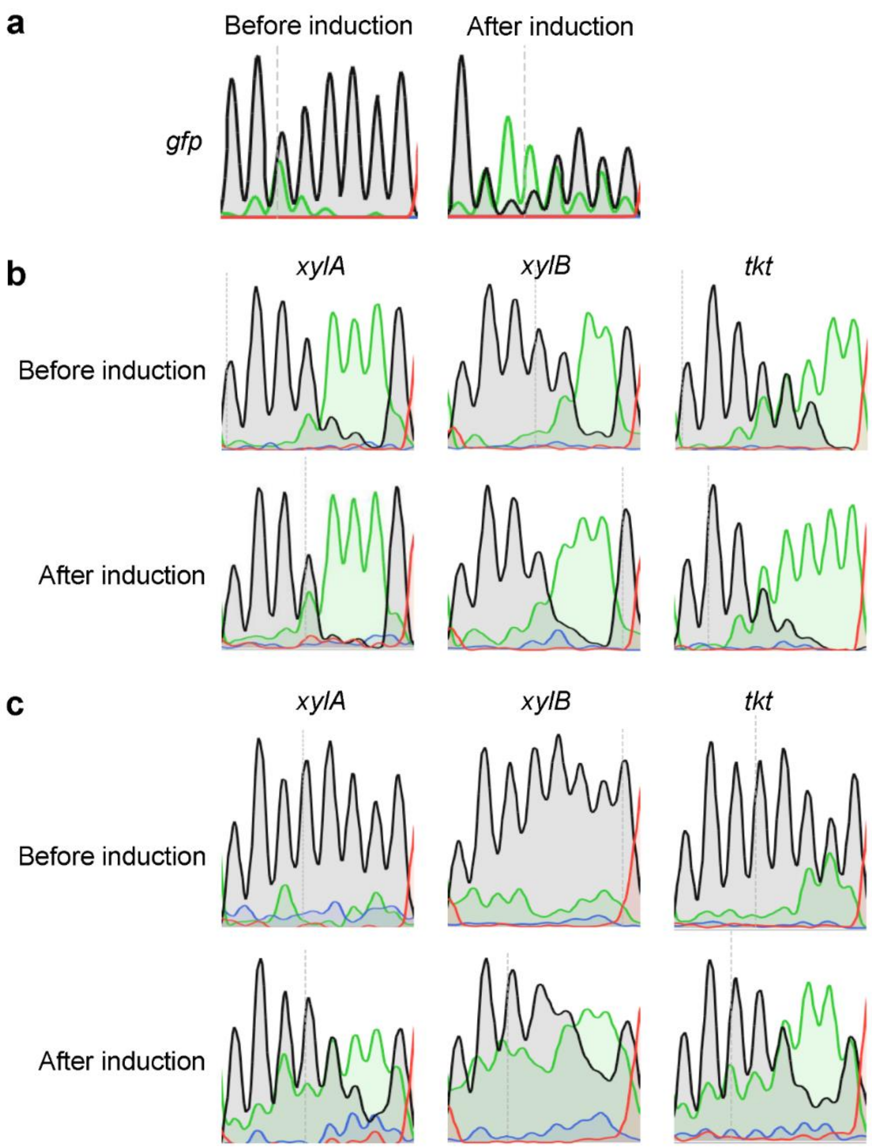

Supplementary Fig. 3. Analysis of base editing on the tailored GGGGGGGG RBS(s) of *gfp* and xylose utilization genes before and after IPTG induction by Sanger sequencing. a, Base editing using pnCas9(D10A)-AID^{TS} and pgRNA-RBS2. nCas9(D10A)-AID was expressed with a strong RBS (GAAAGGAGTTGAGA). Slightly leaky base editing events prior to induction were observed. **b,** Base editing using pnCas9(D10A)-AID^{TS} and pgRNA-RBS2. nCas9(D10A)-AID was expressed with a strong RBS (GAAAGGAGTTGAGA). Severer leaky base editing events prior to induction were observed, leading to bias editing of some nucleotides. **c,** Base editing using pnCas9(D10A)-AID-2^{TS} and pgRNA-RBS2. nCas9(D10A)-AID was expressed with a weaker RBS (GAAAGGCACCCGAT). Weakening translation of nCas9(D10A)-AID led to an overall moderate and average editing of eight Gs. Three clones were used for base editing and the cells were mixed with an equal proportion before extraction of genomic DNAs, PCR amplification, and NGS. A direct comparison of the strength of used RBSs is shown in Supplementary Fig. 4.

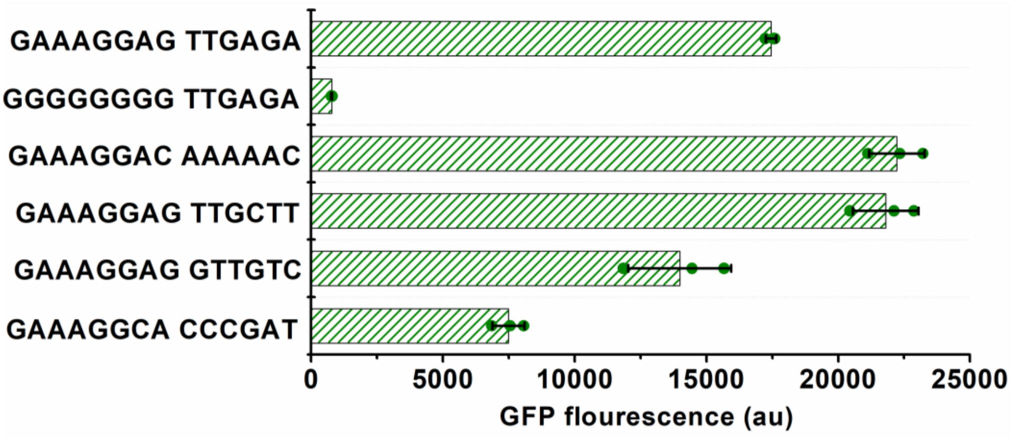

Supplementary Fig. 4. Strength comparison of the RBSs used in this study with previously reported RBSs. GAAAGGAG TTGAGA was used for expressing nCas9(D10A)-AID in plasmid pnCas9(D10A)-AID^{TS} and expressing *gfp* in *C. glutamicum* chromosome as the strong RBS control in Fig. 1d. GGGGGGGG TTGAGA was used as the tailored RBS for constructing RBS libraries using the BETTER method. GAAAGGAC AAAAAC, GAAAGGAG TTGCTT, GAAAGGAG GTTGTC were used as strong RBS controls, which were reported by Zhang et al[1]. GAAAGGCA CCCGAT was also reported by Zhang et al[1] and used for expressing nCas9(D10A)-AID in plasmid pnCas9(D10A)-AID-2^{TS}. RBS strength was determined using a chromosomal GFP reporter. Values and error bars reflect the mean ± s.d. of three biological replicates (n=3). Source data are provided as a Source Data file.

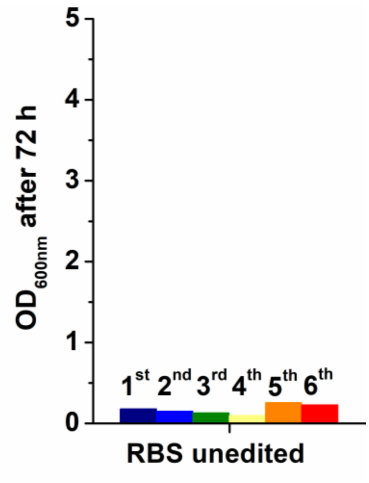

Supplementary Fig. 5. Serial cultivation of the starting strain with *xylA*, *xylB*, and *tkt* controlled by the tailored GGGGGGGG RBS on xylose. Numbers above columns represent the passages of serial cultivation. Source data are provided as a Source Data file.

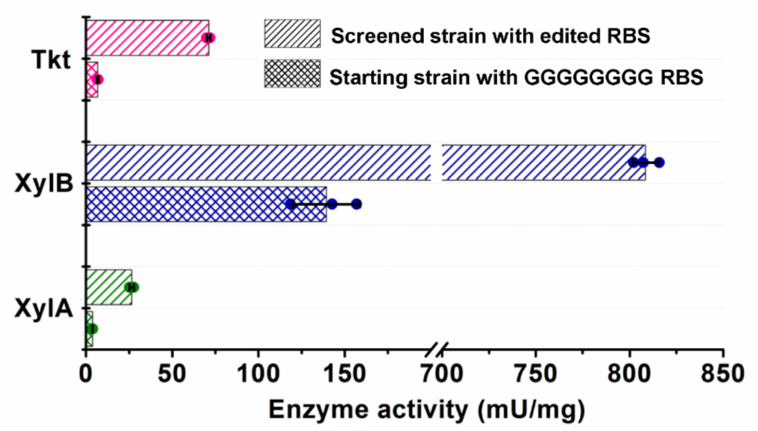

Supplementary Fig. 6. Activities of the xylose utilizing enzymes in the starting strain with GGGGGGGG RBS and the screened strain with edited RBSs. In the starting strain, *xylA*, *xylB*, and *tkt* were controlled by the tailored GGGGGGGG RBS. In the screened strains, *xylA*, *xylB*, and *tkt* were controlled by RBSs GAAAGGAA, AAAAGGAA, and GAAAGGAA, respectively. Values and error bars reflect the mean ± s.d. of three biological replicates (n=3). Source data are provided as a Source Data file.

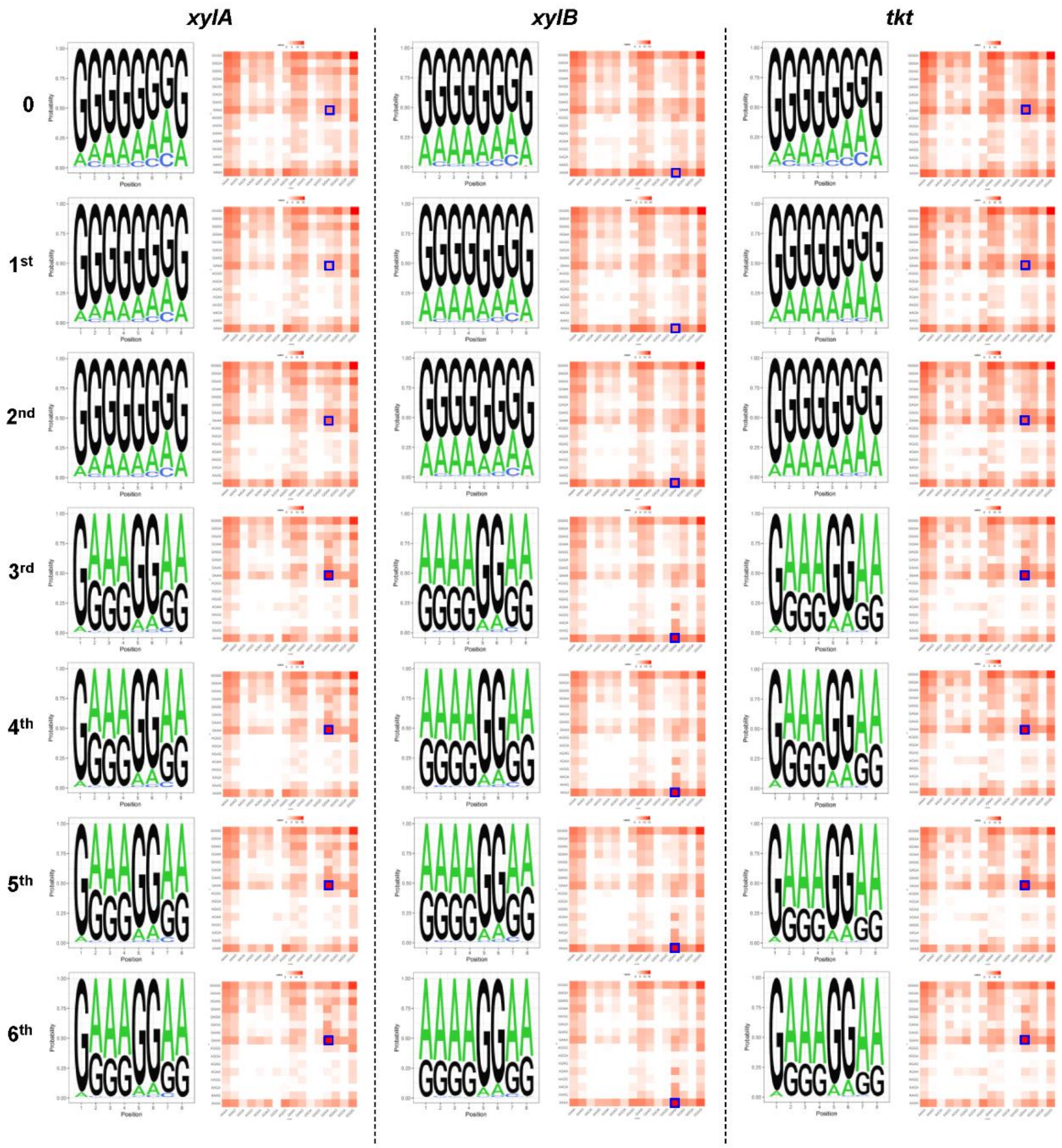

Supplementary Fig. 7. Components of RBS libraries in each passage of serial cultivation in xylose. The matrixes show the coverage and discreteness of 256 G/A-containing RBS variants in the RBS libraries generated by BETTER. Blue squares represent the enriched RBS_{xylA} GAAAGGAA, RBS_{xylB} AAAAGGAA, and RBS_{tkt} GAAAGGAA. Source data are provided as a Source Data file.

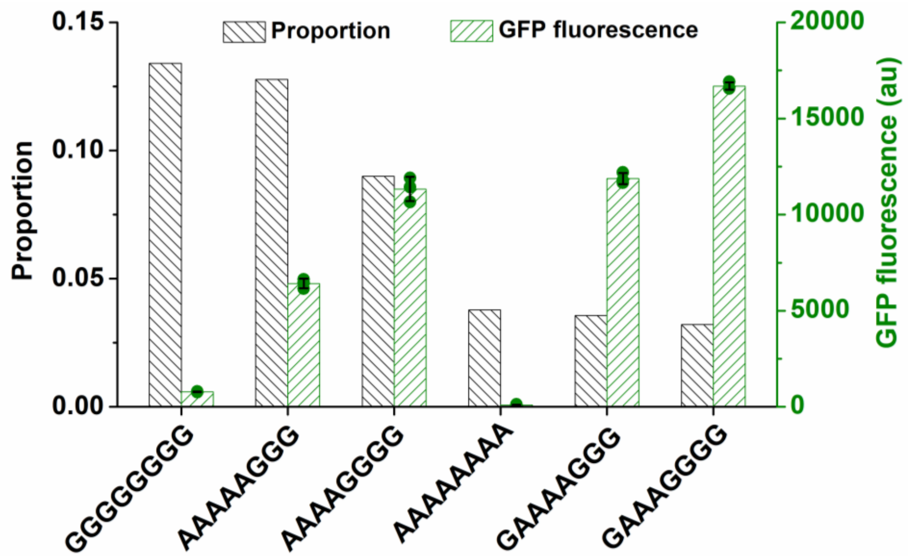

Supplementary Fig. 8. Six RBSs for *crtI* with the largest proportion in the RBS library generated by BETTER. RBS strength was determined using a chromosomal GFP reporter. Values and error bars reflect the mean ± s.d. of three biological replicates (n=3). Source data are provided as a Source Data file.

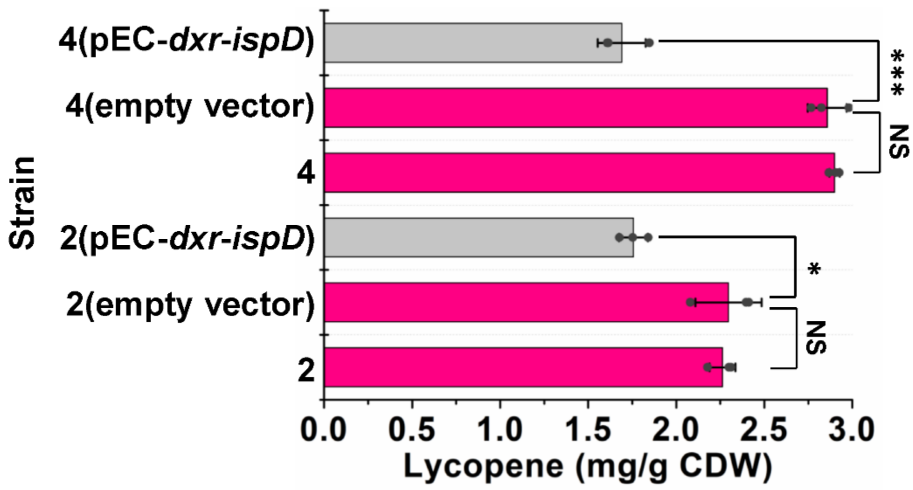

Supplementary Fig. 9. Effects of *dxr* and *ispD* overexpression on lycopene production. Strains 2 and 4 are two variants screened by colorimetric screening. Values and error bars reflect the mean ± s.d. of three biological replicates (n=3). Statistical evaluation (P-value) was performed by two-sided t-test. NS, non-significant ($P \geq 0.05$), *$P<0.05$, ***$P<0.001$, n=3. Source data are provided as a Source Data file.

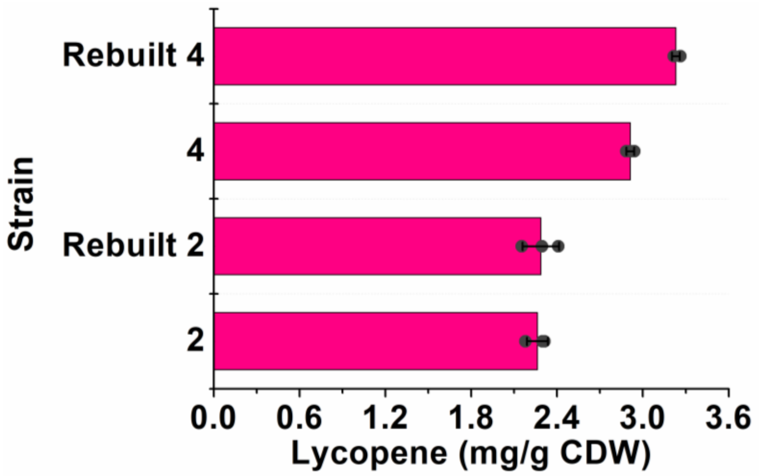

Supplementary Fig. 10. Lycopene production of screened strains (2 and 4) and rebuilt strains (Rebuilt 2 and Rebuilt 4). Strains 2 and 4 are two variants screened by colorimetric screening. Strains Rebuilt 2 and Rebuilt 4 are built according to the RBS combinations of lycopene biosynthesis genes detected in strains 2 and 4. Values and error bars reflect the mean ± s.d. of three biological replicates (n=3). Source data are provided as a Source Data file.

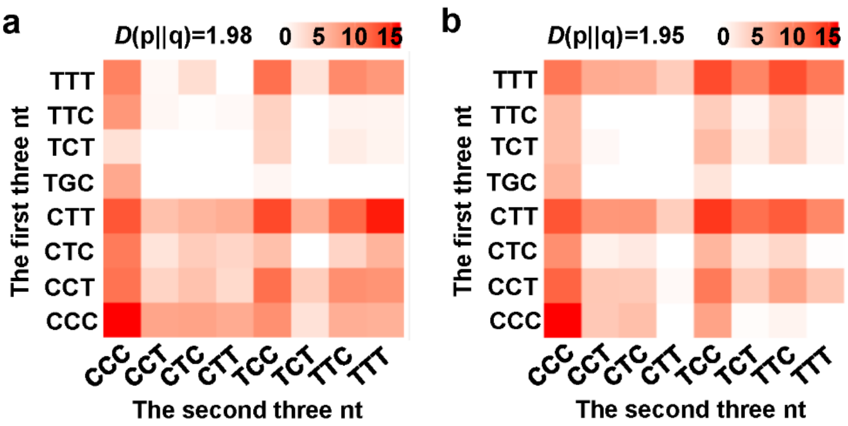

Supplementary Fig. 11. The matrixes showing the coverage and discreteness of 64 C/T-containing variants in the 5'UTR (**a**) and -35 box (**b**) libraries generated by BETTER. KL divergence was used to evaluate the discreteness of genetic combinations. Scale bar represents $\log_2$(relative proportion). Source data are provided as a Source Data file.

a

GCTCCAGTATTCTGACATGGGTGTATCAATAACCCATGCGTTTCCGTATTGTATCGGAATGGTTTCGGACAGG
GCGGTGGGAATAGACATGGAAAAGATTTTTTTGCCCGAGGATGCATTGATAAAGTATTTCTTTTCCGAAAGA
GAGGAAAAGATTCTAAAGAGTTTTGGAAATACTGATGAATATTGTGTGCAGAGTACAATTCTATGGACAAGA
AAAGAGGCTTTGTCAAAACTTTTTCGTCTGGGAATGAGGATGGATTTTAAAAAGCTGGATACTTTGGAGGAC
GAGGTGGTTTTTCAGGAAACAAACAGGGCGCGTCTGTTTTCTTTTATATGCAATAATTACTGTATCTCTCTGG
CATTGCCAGGTTTTAATAAAGATTAAAATTATTGACTAGAAATAAAAAAATTGTCCATAATATTAATGGACAAA
AAAACAAAGAATTACATCAAAGGAAGATAAAAATACTTTGTTAAAAAATTAATTATTTTTTATCTAAACTATTGA
AAATGAAAATAAAATAATATAAAATGAATCATAGTGCAAGAGATACTTGCCAGAGGATGAATATTTTACTGCAT
TCATGCTTTATGGCAGCTAATAGAGGCATTAAACCAACCTTTAATTTACAATAGGAGGCGATATTAATGGGAT
CCATGTCGAAGGGCGAGGAGCTGTTCACCGGCGTCGTCCCGATCCTGGTCGAGCTGGACGGTGACGTCAA
CGGCCACAAGTTCTCCGTCTCCGGCGAGGGTGAGGGCGACGCCACCTACGGCAAGCTGACCCTGAAGTT
CATCTGCACCACCGGTAAGCTGCCGGTCCCGTGGCCGACCCTGGTCACCACCCTGACCTACGGCGTCCAG
TGCTTCTCCCGCTACCCGGACCACATGAAGCGCCACGACTTCTTCAAGTCCGCCATGCCGGAGGGTTACG
TCCAGGAGCGCACCATCTCCTTCAAGGACGACGGTAACTACAAGACGCGTGCCGAGGTCAAGTTCGAGG
GCGACACCCTGGTCAACCGCATCGAGCTGAAGGGCATCGACTTCAAGGAGGACGGTAACATCCTGGGCC
ACAAGCTGGAGTACAACTACAACTCCCACAACGTCTACATCACCGCGGACAAGCAGAAGAACGGCATCAA
GGCCAACTTCAAGACCCGCCACAACATCGAGGACGGTGGCGTCCAGCTAGCCGACCACTACCAGCAGAA
CACCCCGATCGGCGACGGCCCGGTCCTGCTGCCGGACAACCACTACCTGTCCACCCAGTCCGCCCTGTCC
AAGGACCCGAACGAGAAGCGCGACCACATGGTCCTGCTGGAGTTCGTCACCGCCGCCGGCATCACCCAC
GGCATGGACGAGCTGTACAAGTAG

b

GCTCCAGTATTCTGACATGGGTGTATCAATAACCCATGCGTTTCCGTATTGTATCGGAATGGTTTCGGACAGG
GCGGTGGGAATAGACATGGAAAAGATTTTTTTGCCCGAGGATGCATTGATAAAGTATTTCTTTTCCGAAAGA
GAGGAAAAGATTCTAAAGAGTTTTGGAAATACTGATGAATATTGTGTGCAGAGTACAATTCTATGGACAAGA
AAAGAGGCTTTGTCAAAACTTTTTCGTCTGGGAATGAGGATGGATTTTAAAAAGCTGGATACTTTGGAGGAC
GAGGTGGTTTTTCAGGAAACAAACAGGGCGCGTCTGTTTTCTTTTATATGCAATAATTACTGTATCTCTCTGG
CATTGCCAGGTTTTAATAAAGATTAAAATTATTGACTAGAAATAAAAAAATTGTCCATAATATTAATGGACAAA
AAAACAAAGAATTACATCAAAGGAAGATAAAAATACTTTGTTAAAAAATTAATTATTTTTTATCTAAACTATTGA
AAATGAAAATAAAATAATATAAAATGAATCATAGTGCAAGAGATACTTGCCAGAGGATGAATATTTTACTGCAT
TCATGCTTTATGGCAGCTAATAGAGGCATTAAACCAACCTTTAATTTACAATGGGGGGGGATATTAATGGGAT
CCATGTCGAAGGGCGAGGAGCTGTTCACCGGCGTCGTCCCGATCCTGGTCGAGCTGGACGGTGACGTCAA
CGGCCACAAGTTCTCCGTCTCCGGCGAGGGTGAGGGCGACGCCACCTACGGCAAGCTGACCCTGAAGTT
CATCTGCACCACCGGTAAGCTGCCGGTCCCGTGGCCGACCCTGGTCACCACCCTGACCTACGGCGTCCAG
TGCTTCTCCCGCTACCCGGACCACATGAAGCGCCACGACTTCTTCAAGTCCGCCATGCCGGAGGGTTACG
TCCAGGAGCGCACCATCTCCTTCAAGGACGACGGTAACTACAAGACGCGTGCCGAGGTCAAGTTCGAGG
GCGACACCCTGGTCAACCGCATCGAGCTGAAGGGCATCGACTTCAAGGAGGACGGTAACATCCTGGGCC
ACAAGCTGGAGTACAACTACAACTCCCACAACGTCTACATCACCGCGGACAAGCAGAAGAACGGCATCAA
GGCCAACTTCAAGACCCGCCACAACATCGAGGACGGTGGCGTCCAGCTAGCCGACCACTACCAGCAGAA
CACCCCGATCGGCGACGGCCCGGTCCTGCTGCCGGACAACCACTACCTGTCCACCCAGTCCGCCCTGTCC
AAGGACCCGAACGAGAAGCGCGACCACATGGTCCTGCTGGAGTTCGTCACCGCCGCCGGCATCACCCAC
GGCATGGACGAGCTGTACAAGTAG

Supplementary Fig. 12. Sequence of the *gfp* expression cassette used in *B. subtilis*. **a,** The *gfp* expression cassette used as the strong RBS control. **b,** The *gfp* expression cassette with the RBS changed from AGGAGGCG to the tailored GGGGGGGG for editing. Purple fonts, P_{gapDH} promoter; orange fonts, RBS; green fonts, *gfp*.

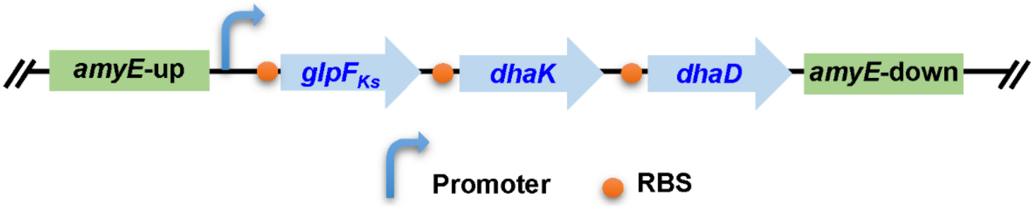

Supplementary Fig. 13. Schematic illustration of integration of heterogeneous glycerol utilizing gene cluster into *B. subtilis* chromosome. The cassette consists of a constitutive promoter P_{gapDH} and three glycerol utilizing genes each with a tailored GGGGGGGG RBS. *glpF*$_{Ks}$, *dhaK*, and *dhaD* are glycerol facilitator, dihydroxyacetone kinase, and glycerol dehydrogenase encoding genes from *Klebsiella* sp. M5a1, respectively.

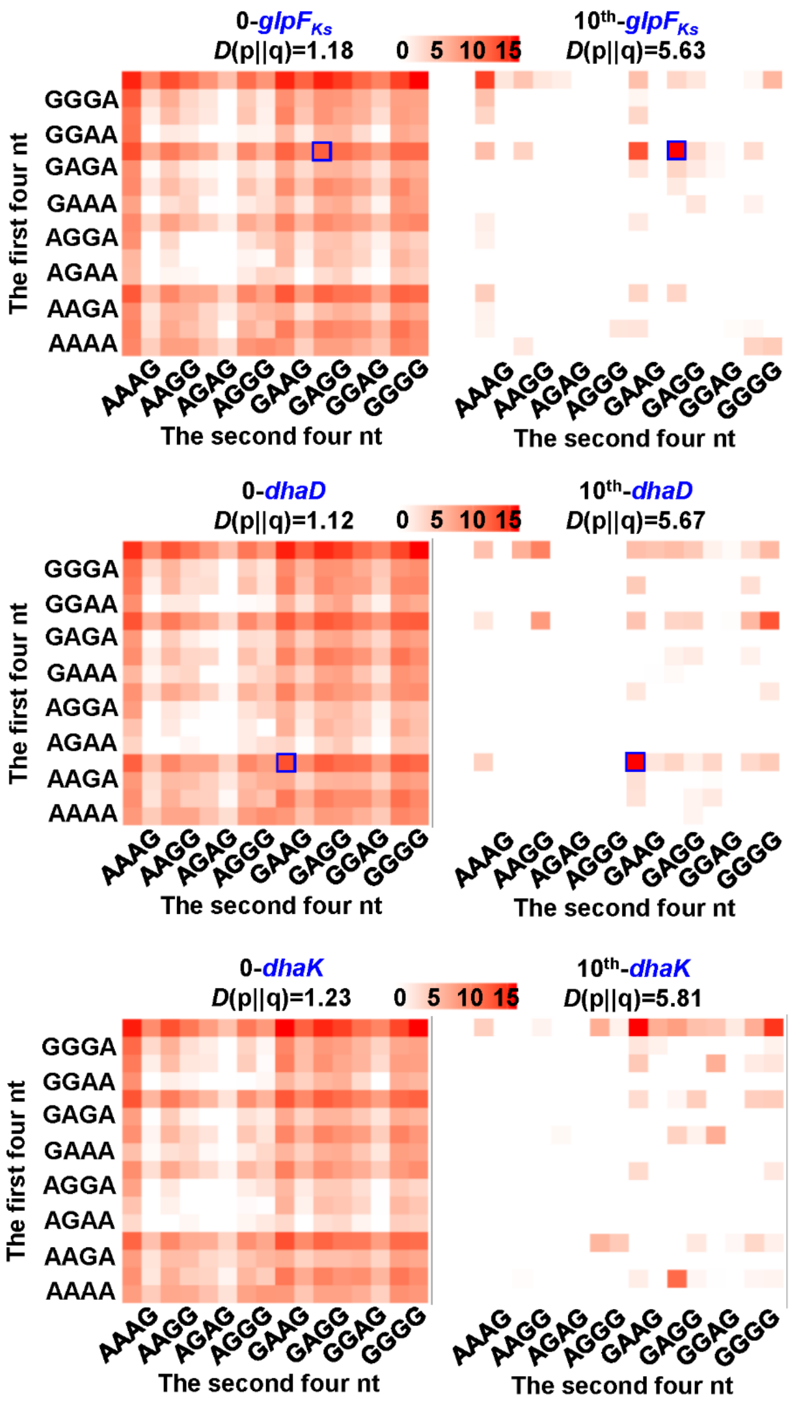

Supplementary Fig. 14. Components of RBS libraries before serial cultivation and after the 10th passage of serial cultivation in glycerol. The matrixes show the coverage and discreteness of 256 G/A-containing RBS variants in the RBS libraries generated by BETTER. Blue squares represent the enriched RBS$_{glpFKs}$ GAGGGAGA and RBS$_{dhaD}$ AAGGGAAA. The enriched RBS$_{dhaK}$ GTAGGGAA contains a T and thus is not shown in the matrixes. Source data are provided as a Source Data file.

Supplementary References

1. Zhang, B. *et al.* Ribosome binding site libraries and pathway modules for shikimic acid synthesis with *Corynebacterium glutamicum*. *Microb. Cell Fact.* **14,** 71 (2015).
