# Supplementary material for: In-situ generation of large numbers of genetic combinations for metabolic reprogramming via CRISPR-guided base editing
Source: Nat Commun. 2021 Jan 29;12:678. doi: 10.1038/s41467-021-21003-y (PMC7846839; doi:10.1038/s41467-021-21003-y)
Supplement: Supplementary file 3 — Reporting Summary [file 41467_2021_21003_MOESM3_ESM.pdf]

nature research | reporting summary

nature research

Corresponding author(s): Meng Wang

Last updated by author(s): Jan 4, 2021

# Reporting Summary

Nature Research wishes to improve the reproducibility of the work that we publish. This form provides structure for consistency and transparency in reporting. For further information on Nature Research policies, see our Editorial Policies and the Editorial Policy Checklist.

## Statistics

For all statistical analyses, confirm that the following items are present in the figure legend, table legend, main text, or Methods section.

| n/a | Confirmed | |
|---|---|---|
| ☐ | ☒ | The exact sample size (*n*) for each experimental group/condition, given as a discrete number and unit of measurement |
| ☐ | ☒ | A statement on whether measurements were taken from distinct samples or whether the same sample was measured repeatedly |
| ☐ | ☒ | The statistical test(s) used AND whether they are one- or two-sided<br>*Only common tests should be described solely by name; describe more complex techniques in the Methods section.* |
| ☒ | ☐ | A description of all covariates tested |
| ☒ | ☐ | A description of any assumptions or corrections, such as tests of normality and adjustment for multiple comparisons |
| ☐ | ☒ | A full description of the statistical parameters including central tendency (e.g. means) or other basic estimates (e.g. regression coefficient) AND variation (e.g. standard deviation) or associated estimates of uncertainty (e.g. confidence intervals) |
| ☐ | ☒ | For null hypothesis testing, the test statistic (e.g. $F$, $t$, $r$) with confidence intervals, effect sizes, degrees of freedom and $P$ value noted<br>*Give P values as exact values whenever suitable.* |
| ☒ | ☐ | For Bayesian analysis, information on the choice of priors and Markov chain Monte Carlo settings |
| ☒ | ☐ | For hierarchical and complex designs, identification of the appropriate level for tests and full reporting of outcomes |
| ☒ | ☐ | Estimates of effect sizes (e.g. Cohen's *d*, Pearson's *r*), indicating how they were calculated |

*Our web collection on statistics for biologists contains articles on many of the points above.*

## Software and code

Policy information about availability of computer code

| | |
|---|---|
| Data collection | 1. Illumina Hiseq Control software was used on the Illumina Hiseq sequencers to collect the sequencing data.<br>2. GFP fluorescence was analyzed by flow cytometry (FACS, Beckman Coulter MoFlo XDP) with the following parameters: excitation at 488 nm, detection fluorescence at 529 ± 14 nm, sample pressure of 60 psi. The nozzle diameter was 70 µm. |
| Data analysis | 1. For NGS data analysis, the 20-nt sequence upstream of the target region was used to locate the position of the target region in each read via blast. Sequence of the target region was then extracted from the read and mapped to the reference sequence to analyze the base editing event. Finally, the number of each RBS variant was counted using the R package (v4.0.2). Sequence logo was generated using the statistical result of G/A/C/T-containing RBS variants and the R package 'ggseqlogo' (v0.1). Heat map was generated using the statistical result of G/A-containing RBS variants and the R package 'ggplot2' (v3.3.2).<br>2. FACS data were analyzed using the Beckman Summit software v5.2.<br>3. All P values were generated from two-tailed t-tests using the Microsoft Excel 2016 (Microsoft Corporation). Kullback-Leible divergence was analyzed using the Microsoft Excel 2016 (Microsoft Corporation).<br>4. Golden Gate assembly protocols were designed using j5 software tool (Revision as of 10:05, 18 October 2018 by J5admin). |

For manuscripts utilizing custom algorithms or software that are central to the research but not yet described in published literature, software must be made available to editors and reviewers. We strongly encourage code deposition in a community repository (e.g. GitHub). See the Nature Research guidelines for submitting code & software for further information.

## Data

Policy information about availability of data

All manuscripts must include a data availability statement. This statement should provide the following information, where applicable:

- Accession codes, unique identifiers, or web links for publicly available datasets
- A list of figures that have associated raw data
- A description of any restrictions on data availability

The raw reads of the NGS data were deposited into the Sequence Read Archive (SRA) database (accession number: PRJNA608771) at the National Center for Biotechnology Information (NCBI). Data supporting the findings of this work are available within the paper and its Supplementary Information files. A reporting summary for this Article is available as a Supplementary Information file. The datasets and materials generated and analyzed during the current study are available from the corresponding author upon request. The source data underlying Figures 1c, 2c-h, 3d, 3e, 3g, 5c, 5e, 5g, 5h, as well as Supplementary Figures 4, 5, 6, 7, 8, 9, 10, 11, and 14 are provided as a Source Data file.

# Field-specific reporting

Please select the one below that is the best fit for your research. If you are not sure, read the appropriate sections before making your selection.

- [x] Life sciences
- [ ] Behavioural & social sciences
- [ ] Ecological, evolutionary & environmental sciences

For a reference copy of the document with all sections, see nature.com/documents/nr-reporting-summary-flat.pdf

# Life sciences study design

All studies must disclose on these points even when the disclosure is negative.

| | |
|---|---|
| Sample size | The sample size of experiments is determined at 3 (biological independent replicate) based on literature precedence for metabolic engineering experiments (Ref. https://doi.org/10.1038/s41467-020-16962-7; https://doi.org/10.1038/s41467-020-17223-3). |
| Data exclusions | No data were excluded from the analyses. |
| Replication | All experiments were repeated at least once. All attempts at replication were successful. |
| Randomization | E. coli, C. glutamicum, and B. subtilis strains used in this study were grown under identical conditions; no randomization was used. |
| Blinding | Blinding was not performed in the study because the experiments did not involve any animals or human participants. The analysis was carried out entirely on bacteria. |

# Reporting for specific materials, systems and methods

We require information from authors about some types of materials, experimental systems and methods used in many studies. Here, indicate whether each material, system or method listed is relevant to your study. If you are not sure if a list item applies to your research, read the appropriate section before selecting a response.

## Materials & experimental systems

| n/a | Involved in the study | |
|---|---|---|
| x | | Antibodies |
| x | | Eukaryotic cell lines |
| x | | Palaeontology and archaeology |
| x | | Animals and other organisms |
| x | | Human research participants |
| x | | Clinical data |
| x | | Dual use research of concern |

## Methods

| n/a | Involved in the study | |
|---|---|---|
| x | | ChIP-seq |
| | x | Flow cytometry |
| x | | MRI-based neuroimaging |

## Flow Cytometry

### Plots

Confirm that:

☒ The axis labels state the marker and fluorochrome used (e.g. CD4-FITC).

☒ The axis scales are clearly visible. Include numbers along axes only for bottom left plot of group (a 'group' is an analysis of identical markers).

☒ All plots are contour plots with outliers or pseudocolor plots.

☒ A numerical value for number of cells or percentage (with statistics) is provided.

### Methodology

| | |
|---|---|
| Sample preparation | Cells were harvested from cultures by centrifugation at 6,000 × g for 10 min, washed once, and re-suspended in phosphate buffer (pH 7.4). |
| Instrument | FACS, Beckman Coulter MoFlo XDP |
| Software | Beckman Summit software v5.2 |
| Cell population abundance | One hundred thousand C. glutamicum cells were analyzed. |
| Gating strategy | GFP fluorescence was analyzed by flow cytometry (FACS, Beckman Coulter MoFlo XDP) with the following parameters: excitation at 488 nm, emission fluorescence at 529 ± 14 nm, sample pressure of 60 psi. The nozzle diameter was 70 µm. Cells were captured on the signal channels of FITC (voltage 510 V), FSC (voltage 150 V) and SSC (voltage 250 V). All captured events were used for fluorescence analysis. |

☒ Tick this box to confirm that a figure exemplifying the gating strategy is provided in the Supplementary Information.
