# Supplementary material for: In-situ generation of large numbers of genetic combinations for metabolic reprogramming via CRISPR-guided base editing
Source: Nat Commun. 2021 Jan 29;12:678. doi: 10.1038/s41467-021-21003-y (PMC7846839; doi:10.1038/s41467-021-21003-y)
Supplement: Supplementary file 4 — Description of Additional Supplementary Files [file 41467_2021_21003_MOESM4_ESM.pdf]

Description of Additional Supplementary Files

Supplementary Data 1

RBS variants generated by BETTER with a single 20-nt gRNA or a double 20-nt gRNAs combination.

Supplementary Data 2

RBS variants generated by BETTER and changes during six passages of serial cultivation.

Supplementary Data 3

Enrichment of specific RBS variants during six passages of serial cultivation.

Supplementary Data 4

RBS variants generated by BETTER with different combinations of double gRNAs.

Supplementary Data 5

RBS of ten target genes in screened lycopene producers.

Supplementary Data 6

Variants of 5‘UTR and -35 box generated by BETTER with a single 20-nt gRNA.

Supplementary Data 7

RBS variants generated by BETTER with a double 20-nt gRNAs combination in B. subtilis.

Supplementary Data 8

RBS variants of glycerol pathway genes generated by BETTER.

Supplementary Data 9

Strains and plasmids used in this study.

Supplementary Data 10

Primers and ssDNAs used in this study.
