# Supplementary material for: In-situ generation of large numbers of genetic combinations for metabolic reprogramming via CRISPR-guided base editing
Source: Nat Commun. 2021 Jan 29;12:678. doi: 10.1038/s41467-021-21003-y (PMC7846839; doi:10.1038/s41467-021-21003-y)
Supplement: Supplementary file 2 — Peer Review File [file 41467_2021_21003_MOESM2_ESM.pdf]

Reviewer #1 (Remarks to the Author):

This manuscript describes about localized gene expression control by base editing at tailored Ribosome Binding site (RBS) using CRISPR based method called Base Editor Targeted and Template-free Expression Regultion (BETTER). This method can produce a large number of RBS variants leading to fine tuning of gene expression. This method will be useful to identify optimal RBS varients for each gene in a pathway and improve the product titre in an industrially relavant strains of bacteria that is somewhat difficult to transform. The technology is demonstrated on xylose metabolism and lycopene production. While the technology is potentially of interest to those working in a wide number of strains, the two examples demonstrated convey unimpressive results. For example, the lycopene yield falls an order of magnitude shy of the work by Xu et al., 2018 Bioprocess Biosyst Eng and is not improved by a large amount from the work cited in Ref 8. Therefore, I do not consider this work impactful enough for publication in Nature Communications.

Questions
1. The statement that almost infinite genetic combinations (2.7x1033) were produced for ten target genes is incorrect. The actual size of the library should be measured by illumina sequencing.
2. Author had compared the Screened strain and Built strain in Xylose Utilization experiment. But, In Fig 3, lycopene experiment, author not build the pathway using identified optimal RBS varients and not compared with the BETTER screened strain. This will be needed to demonstrate the RBS engineering explains the phenotype.
3. In this study, xylA is the only heterologous gene introduced in the Corynebacterium glutamicum, all others are native genes. Is there any expression difference observed with native and heterologous gene by using tailored RBS based BETTER method?
4. Can author explain the rationale behind choosing eight consecutive G's as a low efficiency RBS using references?
5. In page 4, line 70 author claims "lack of efficient recombination pathways available in C. glutamicum". This arguments is not entirely correct. Good progress has been made in recent years. Examples, Zhang, J., et al. Optimizing a CRISPR-Cpf1-based genome engineering system for Corynebacterium glutamicum. Liu, W, et al. Combined genome editing and transcriptional repression for metabolic pathway engineering in Corynebacterium glutamicum using a catalytically active Cas12a.
6. There is no control used in Figure 2 (g) for xylose utilization. It will be good if author can show us the xylose utilization rate from the strain expressing xylA, tkt, xylB contains taliored RBS sequence, but without base editing. This could provide the expression variations between BETTER method vs direct evolution method in 6th passage
7. In Fig. 2 (c) author stops at 6th passage? Will the OD600 be further improved after 6th passage? Will the dominant RBS varients remains the same after 6th passage?
8. In Fig. 2 (g) the built strain growth declined after 20h but, the screened strain reaches stationary phase at 24h. Can author explain the possible reason for the difference?
9. Can author reason out why the overexpression of dxr and ispD decreased lycopene titre? Will lycopene titer be improved if heterologous dxr and ispD introduced in the cells rather than alterting native RBS region?
10. In page 18, line 355 replace the word from "detection" to "emission"
11. RBS spelling error at Page 15, line 292

Reviewer #2 (Remarks to the Author):

In this manuscript titled "In-situ generation of almost infinite genetic combinations for 3 metabolic reprogramming via CRISPR-guided base editing", the authors devised a targeted base-editing tool named BETTER and enabled rapid in situ generation of RBS libraries containing huge number of variants. Applying BETTER to Corynebacterium glutamicum, the authors generated strains utilizing xylose for growth or producing lycopene from glucose at noticeable performances, demonstrating

great efficiency of this tool in generating RBS libraries. This manuscript was nicely written with well-designed experiments and thorough analysis of the experimental data. However, some numbers and expressions are exaggerated. In addition, more examples are expected to emphasize broad applicability of this efficient system. Additional set of experiments and rewording some sentences would upgrade the quality of this manuscript.

Major comments
Title and manuscript: The title should be changed so that it reflects what they have actually done, rather than overly stating. Here, the authors have used nCas9-cystidine deaminase for the generation of large RBS libraries for up to 10 genes for the production of lycopene. It seems that this system may be useful only for the production of chemicals that can be screened using colorimetric assays. Also, for the authors to justify their title, they must examples of other genetic combinations other than RBS libraries. For example, they should have also shown promoter libraries, UTR libraries, terminator libraries, gene mutation libraries to encompass genetic combinations.
Throughout the manuscript: Some RBS sequences were mentioned strong or weak without appropriate data or references. Please provide evidences for their strength.
Line 71-73: The distance between an RBS and a start codon affects the expression strength. However, the exact location of the tailored GGGGGGGG sequence before the RBS library generation was not mentioned. Please specify a clear rule of determining the location of GGGGGGGG introduction relative to the location of a start codon.
Line 81-82: Please provide total number of sequence reads examined to detect 94 and 202 variants, respectively. This information is critical to estimate the library-generating efficiency of BETTER.
Line 85-87: Please provide total number of reads examined to identify 527 and 970 G/A/C/T-containing RBS variants, respectively.
Line 87-88: The expression "1,000-fold dynamic range of GFP expression" is ambiguous. Please consider rewording.
Line 116-118: Please explain the reason why 'the unexpected base editing before induction' did not occur while modifying the RBS of the gfp gene.
Line 121-124: Again, please inform in this sentence the total number of variant population examined for each experimental set.
Line 168-175: It has been well reported that the translation efficiency depends not on only on RBS sequence but also on the sequence of the 5′ region of CDS. Thus, the enhancement of RBS strength measured using the GFP reporter system may not represent the actual RBS strength for the xylose utilization genes.
Line 229-231: 'Almost infinite' is an exaggeration since 2.7×1033 is definitely a finite number. In addition, the actual number of variants in the library was overestimated by simply multiplying library sizes of each gene – many combinations of variants would not exist in the library unless the total cell population was much larger than 1033 (~105 mol of cells). Please provide the size of population examined to identify such a large number of diversity.
Line 233-235: Examination of only 105 cells out of a huge library is a big waste of resources. Examination of larger population with high-throughput screening devices would be helpful to find strains with better performances.
Line 292-294: BETTER does seem so simple and efficient. The easiness and general applicability of BETTER would be greatly emphasized if additional examples in other microorganisms are included in this manuscript. Please apply BETTER to microorganisms other than C. glutamicum (e.g. Escherichia coli and Saccharomyces cerevisiae) and provide a couple of examples of improving strain performances. Otherwise, please confine the title to Corynebacterium glutamicum.

Minor comments
Line 28: Consider changing the verb form 'regulating' to 'regulate'.
Line 31: Suggest changing the word "shall" to "will"
Line 37: 'burden' must be corrected into 'burdens'.
Line 54: 'notoriously' Suggest using a less subjective word.
Line 57-58: BETTER requires tailored RBS sequences with eight consecutive Gs, which requires DNA

template-dependent engineering. Thus, “in a DNA template-free and DNA incorporation-independent manner” is incorrect. Please consider correcting the sentence.
Line 163: Please provide quantitative kinetic data of the fastest xylose uptake rate in C. glutamicum. to make the comparison easier.
Line 174: Change ‘elevated’ to either ‘the elevated’ or ‘an elevated’.
Line 187-188: This sentence sounds weird. Please proofread this sentence.
Line 253: Please correct ‘C. gultamicum’ to ‘C. glutamicum’
Line 255: Supplementary Fig. 5 should be cited instead of Supplementary Fig. 4.
Line 278: ‘astonishing’ Suggest using a less subjective word.
Line 306: ‘.’ is missing at the end of the sentence.

Responses to reviewers' comments point-by-point

Thank you for the reviewers' critical comments and thoughtful suggestions, which are very helpful for us to improve our manuscript. We carefully considered all the comments and revised the manuscript accordingly. The revised manuscript with the changes highlighted in red for your convenience has been uploaded through the online submission system. We hope that the answers and the changes in the manuscript will enable our manuscript to win your satisfaction. The point-by-point response to your comments are listed below.

Reviewer #1 (Remarks to the Author):

This manuscript describes about localized gene expression control by base editing at tailored Ribosome Binding site (RBS) using CRISPR based method called Base Editor Targeted and Template-free Expression Regultion (BETTER). This method can produce a large number of RBS variants leading to fine tuning of gene expression. This method will be useful to identify optimal RBS varients for each gene in a pathway and improve the product titre in an industrially relavant strains of bacteria that is somewhat difficult to transform. The technology is demonstrated on xylose metabolism and lycopene production. While the technology is potentially of interest to those working in a wide number of strains, the two examples demonstrated convey unimpressive results. For example, the lycopene yield falls an order of magnitude shy of the work by Xu et al., 2018 Bioprocess Biosyst Eng and is not improved by a large amount from the work cited in Ref 8. Therefore, I do not consider this work impactful enough for publication in Nature Communications.

Response:

Thank you for your helpful comments. We would like to discuss with you about the demonstration of BETTER technology. We find the paper you mentioned (Xu et al., 2018 Bioprocess Biosyst Eng) is about lycopene production in *E. coli*, which is a research topic with decades of history and hundreds of papers. It seems unfair to directly compare lycopene titer in *E. coli* to *C. glutamicum*, in which only a handful of research papers related to lycopene production was published. After all, lycopene production is not only dependent on expression levels of the ten genes we studied in our paper. According to the paper you mentioned, many other factors would have great impact on lycopene production, such as the competition of byproducts pathways,

supply of precursor, medium components, and fermentation operation strategy. In addition, we think our main novelty is that our method can easily create a large combinatorial library on ten genomic sites, which currently can't be done in *C. glutamicum* and most other microorganisms. Titer improvement is just a demonstration but not our ultimate goal here. If we screen more clones, we can find strains with improved titers, but it doesn't seem to be very meaningful for the elevation of the novelty of our method.

However, to address your concern, we did additional experiments to demonstrate the general applicability of this BETTER method in RBS, promoter, and 5'UTR diversification, in not only *C. glutamicum* but also another industrially important chassis and model microbe *Bacillus subtilis*. We hope the additional data supplemented in the revision can enable our manuscript to win your satisfaction.

The newly added data have been added in the revised manuscript and are summarized as follows.

1. Application of BETTER in diversifying 5' untranslated region (5' UTR) and promoter.

To expand the application of BETTER in diversifying other translation or transcription elements, we tested library construction for the 5'UTR between RBS and the start codon and the –35 box of promoter. Starting from a CCCCCC 5'UTR or -35 box, BETTER generated a C/T-rich library. All the 64 C/T-containing combinations were detected by NGS. Totally 772 and 900 out of 4,096 kinds of G/A/C/T-containing combinations were detected for 5'UTR and -35 box, respectively (Supplementary Table 6). By analyzing GFP expression using flow cytometry, it was found that the edited cells showed diverse GFP fluorescence signals (Figs. 4b and 4c). The results suggest that the BETTER method can also be used to diversify gene expression by generating libraries for other translation or transcription elements than RBS, such as 5'UTR and promoter.

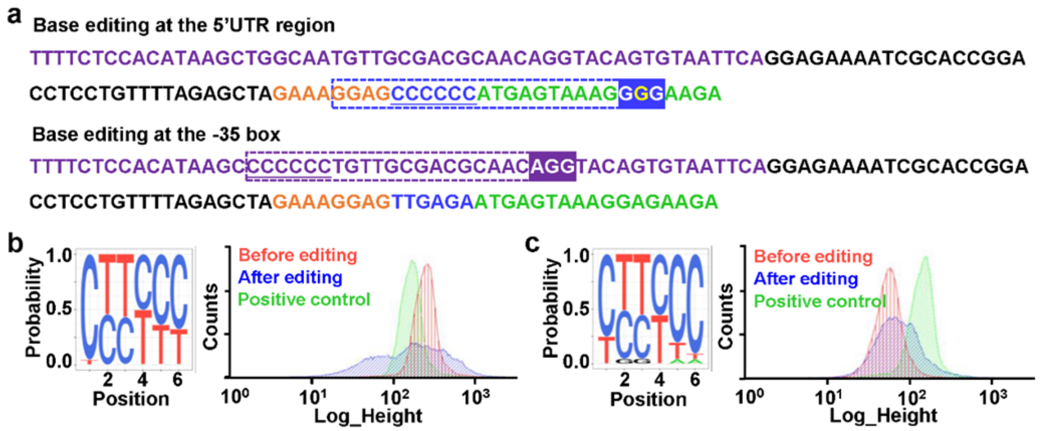

Fig. 4 Diversifying 5'UTR and promoter by BETTER for regulating gene expression. a, Design of base editing targets by replacing the original 5'UTR between RBS and start codon and –35 box of promoter to six consecutive Cs, respectively. The target Cs are underlined. The N20 sequences of gRNA are denoted with the dotted box. PAMs for 5'UTR and promoter editing are shaded in blue and purple, respectively. The mutated G for generation of a GGG PAM is highlighted in yellow. The original sequence for *gfp* expression cassette is shown Supplementary Fig. 1. **b**, **c**, Analyses of the 5'UTR (**b**) and –35 box (**c**) libraries by NGS and flow cytometry. Three colonies were used for base editing and the cells were mixed with an equal proportion before extraction of genomic DNAs, PCR amplification, and NGS. The original *gfp* expression cassette was used as a positive control.

2. Application of BETTER in *B. subtilis*.

In order to further verify the general applicability of the BETTER method for microorganisms other than *C. glutamicum*, the method was expanded to another model bacterium *B. subtilis*, in which we have recently developed an efficient base editing technology. GFP was first used as a reporter to test the BETTER method for construction of RBS library. The strength of generated G/A-rich RBS library spanned nearly three orders of magnitude in terms of GFP fluorescence intensity (Fig. 5b), demonstrating the good performance of BETTER in *B. subtilis*. A detailed analysis reveals the generation of 230 out of 256 G/A-containing RBS variants with a KL divergence value of 1.87 (Fig. 5c), and the number increases to 3,519 in the case of calculating G/A/C/T-containing RBS variants (Supplementary Table 7).

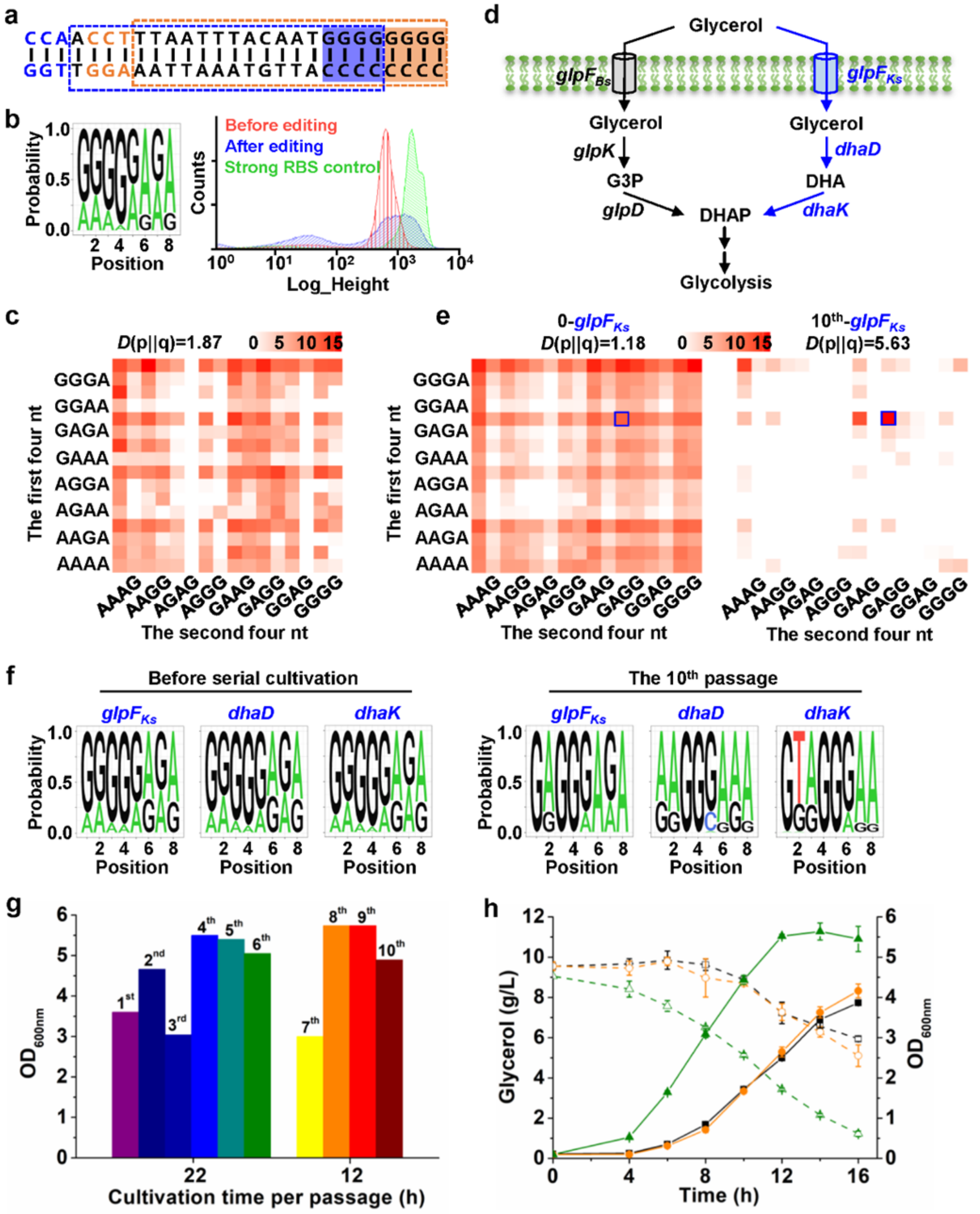

Fig. 5 Regulating *gfp* expression and reprogramming glycerol catabolism by BETTER in *B. subtilis*. a, Two interlaced gRNAs facilitate editing of eight Gs. A set of PAM, gRNA, and editing window is shown in the same color. **b**, Analyses of the RBS_{gfp} library by NGS and flow cytometry. Three colonies were used for base editing and the cells were mixed with an equal proportion before extraction of genomic DNAs, PCR amplification, and NGS. A strong RBS (AGGAGGCG)[34] was used as a control. **c**, The matrix shows the coverage and discreteness of 256 G/A-containing RBS variants in the RBS_{gfp} library generated by BETTER with double gRNAs. Scale

bar represents $\log_2$(relative proportion). KL divergence ($D(p \| q)$) was used to evaluate the discreteness of genetic combinations. **d**, The native glycerol catabolism pathway of *B. subtilis* (black arrows) and the heterologous pathway from *Klebsiella* sp. (blue arrows). G3P, glyceraldehyde 3-phosphate; DHA, dihydroxyacetone; DHAP, dihydroxyacetone phosphate. $glpF_{Bs}$, glycerol facilitator from *B. subtilis*; *glpK*, glycerol kinase; *glpD*, G3P dehydrogenase; $glpF_{Ks}$, glycerol facilitator from *Klebsiella* sp.; *dhaD*, glycerol dehydrogenase; *dhaK*, dihydroxyacetone kinase. **e**, The matrixes show the coverage and discreteness of 256 G/A-containing RBS variants in the RBS libraries of $glpF_{Ks}$ generated by BETTER before (0) and after ten passages (10th) of serial cultivation in glycerol. Scale bar represents $\log_2$(relative proportion). Blue squares represent the enriched RBS_{glpFKs} GAGGGAGA. The matrixes for RBS libraries of *dhaD* and *dhaK* are shown in Supplementary Fig. 14. Three colonies were used for base editing and the cells were mixed with an equal proportion before extraction of genomic DNAs, PCR amplification, and NGS. **f**, Components of RBS libraries before and after ten passages of serial cultivation in glycerol. **g**, Serial cultivation for screening genetic combinations that favor growth on glycerol. Numbers above columns represent the passages of serial cultivation. For the first six passages, transfer was made after 22 h cultivation. For the subsequent four passages, the cultivation time for each passage was reduced to 12 h because cell growth rate largely increased. **h**, Growth (filled symbols) and glycerol consumption (open symbols) of wild-type *B. subtilis* 168 (black square), the starting strain (orange circle) with heterologous $glpF_{Ks}$, *dhaD*, and *dhaK* genes controlled by GGGGGGGG RBSs, and the screened strain (green triangle) from the 10th passage with RBS_{glpFKs} GAGGGAGA, RBS_{dhaD} AAGGGAAA, and RBS_{dhaK} GTAGGGAA. Error bars indicate standard deviations from three parallel experiments.

Next, we reprogramed the glycerol catabolism pathway to accelerate glycerol utilization in *B. subtilis*. Glycerol is a major by-product of biodiesel production and a promising feedstock for biomanufacturing. It also enhances bioproduction of some valuable chemicals (e.g. menaquinone-7 and uridine) when used as the carbon source for *B. subtilis*. An artificial cluster consisting of a constitutive promoter P_{gapDH}, $glpF_{Ks}$ (encoding glycerol facilitator), *dhaD* (encoding glycerol dehydrogenase), and *dhaK* (encoding dihydroxyacetone kinase) from an efficient glycerol utilizer *Klebsiella* sp. was integrated into the *B. subtilis* 168 chromosome (Fig. 5d). BETTER generated

good RBS libraries for three genes (Figs. 5e and 5f and Supplementary Table 8). The edited cell mixture was serially cultivated in M9 minimal medium with glycerol as a sole carbon source and accelerated growth rate was observed (Fig. 5g). NGS analysis shows that RBS_{glpFKs} GAGGGAGA, RBS_{dhaD} AAGGGAAA, and RBS_{dhaK} GTAGGGAA were enriched in varying degrees (Fig. 5f and Supplementary Fig. 14). The growth rate and glycerol uptake rate of the screened strain were apparently higher than those of the controls (Fig. 5h). The improvement demonstrates the applicability of BETTER in metabolic engineering of *B. subtilis*.

Questions

1. The statement that almost infinite genetic combinations ($2.7x10^{33}$) were produced for ten target genes is incorrect. The actual size of the library should be measured by illumina sequencing.

Response:

Thank you for your helpful comments. We realized that the statement of library size was misleading and modified it in the revised manuscript. For illumina sequencing, $\sim10^9$ cells were collected and used for genomic DNA extraction. The genomic DNA was then used as a template to amplify the base editing region and the amplicon was sequenced. Previously stated library size of $2.7x10^{33}$ is an estimated theoretically maximum library complexity based on sequenced RBS diversities of individual sites. It is merely a mathematical projection and never means to represent the actual size of our library. In addition, the exact number of genetic combinations in our library cannot be determined even via the most advanced sequencing technologies. Ten RBS loci are scattered on different regions of the genome, and the only way we think might be able to determine a genotype is single-cell genome sequencing. However, performing single-cell genome sequencing on such a large population of cells is impractical and seems unnecessary for the present study.

Please refer to Page 15, Line 279 for the revised statement: "Targeted sequencing revealed the G/A/C/T-containing RBS types were 3,550 for *dxs* (including 237 G/A-containing types), 1,873 for *dxr* (including 221 G/A-containing types), 1,847 for *ispD* (including 238 G/A-containing types), 3,061 for *ispE* (including 237 G/A-containing types), 1,694 for *ispF* (including 237 G/A-containing types), 1,666 for *ispG* (including 223 G/A-containing types), 1,987 for *ispH* (including 236 G/A-containing types), 3,386 for *crtE* (including 245 G/A-containing types), 2,263 for

crtB (including 236 G/A-containing types), and 1,667 for *crtI* (including 231 G/A-containing types) (Supplementary Table 4). It was estimated that the theoretically maximum library complexity could reach 2.7×10^{33} if a large enough population of cells were examined."

2. Author had compared the Screened strain and Built strain in Xylose Utilization experiment. But, In Fig 3, lycopene experiment, author not build the pathway using identified optimal RBS variants and not compared with the BETTER screened strain. This will be needed to demonstrate the RBS engineering explains the phenotype.

Response

Thanks for your helpful comments. We have rebuilt two lycopene producing strains from the wild-type strain using the screened RBS variants. The screened strain and built strain showed similar lycopene production. The data have been added in the revised manuscript as Supplementary Fig. 10.

Please refer to Page 16, Line 316: "To verify the improvement in lycopene biosynthesis is solely due to RBS editing, we rebuilt the strains 2 and 4 from the wild-type strain. Three rounds of homologous recombination were conducted to insert three artificial clusters with the edited RBS combinations. The rebuilt strain 2 showed almost the same lycopene production capability with the screened strain 2, while the rebuilt strain 4 produced slightly more lycopene than the screened strain 4 (Supplementary Fig. 10). The results demonstrate that the RBS engineering dominantly leads to the phenotype change."

Please refer to Supplementary Fig. 10:

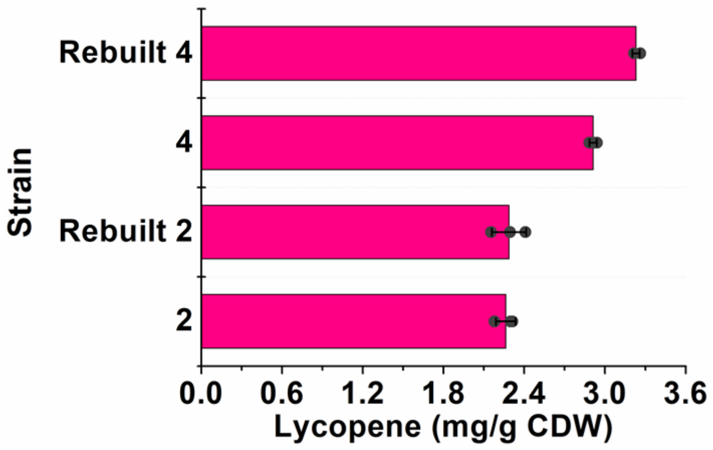

Supplementary Fig. 10 Lycopene production of screened strains (2 and 4) and rebuilt strains (Rebuilt 2 and Rebuilt 4). Strains 2 and 4 are two variants screened

by colorimetric screening. Strains Rebuilt 2 and Rebuilt 4 are built according to the RBS combinations of lycopene biosynthesis genes detected in strains 2 and 4. Error bars indicate standard deviations from three parallel experiments.

3. In this study, xylA is the only heterologous gene introduced in the Corynebacterium glutamicum, all others are native genes. Is there any expression difference observed with native and heterologous gene by using tailored RBS based BETTER method?

Response

Thanks for your helpful comments. In BETTER application in *C. glutamicum*, *xylA* from *E. coli* and the reporter gene *gfp* are heterologous genes. In BETTER application in *B. subtilis*, *gfp*, *glpF*$_{Ks}$, *dhaD*, and *dhaK* are all heterologous genes. Based on our results, no significant difference in BETTER application was observed for native and heterologous genes.

Generally, there might be some problems for expression of heterologous genes because of codon usage bias. The sequence of the 5' region of CDS is also suggested to affect the translation efficiency. Codon optimization should help in this regard. Here, a codon usage analysis using the JCat tool (Nucleic Acids Res. 33, W526–531 (2005)) shows that both *xylA* and *gfp* have many rare codons for expression in *C. glutamicum* (Figs. R1 and R2). However, these two genes were normally expressed in our experiment. As shown in Fig. 1d, the strength of generated RBS variants spanned nearly three orders of magnitude in terms of GFP fluorescence intensity. We also detected the activities of xylose utilizing enzymes in the crude cell extract of the starting strain with GGGGGGGG RBSs and the screened strain with edited RBSs. As shown in Supplementary Fig. 6, the XylA activity in the screened strain (RBS$_{xylA}$ GAAAGGAA) is 7-fold higher than that in the starting strain (RBS$_{xylA}$ GGGGGGGG). The result suggests *xylA* expression is largely enhanced even it is a heterologous gene. We have added discussion on the effects of heterologous genes on application of the BETTER method.

Please refer to Page 25, Line 504:"Codon optimization can also be conducted for heterologous genes during DNA synthesis to overcome the potentially negative effects of rare codons on gene expression."

Please refer to Supplementary Fig. 6:

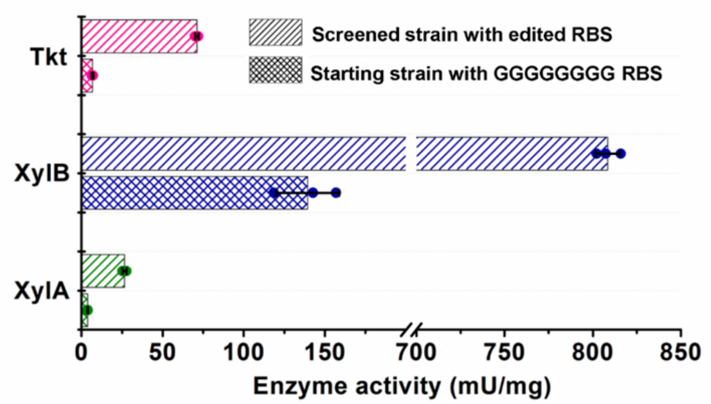

Supplementary Fig. 6 Activities of the xylose utilizing enzymes in the starting strain with GGGGGGGG RBS and the screened strain with edited RBSs. In the starting strain, *xylA*, *xylB*, and *tkt* were controlled by the tailored GGGGGGGG RBS. In the screened strains, *xylA*, *xylB*, and *tkt* were controlled by RBSs GAAAGGAA, AAAAGGAA, and GAAAGGAA, respectively. Error bars indicate standard deviations from three parallel experiments.

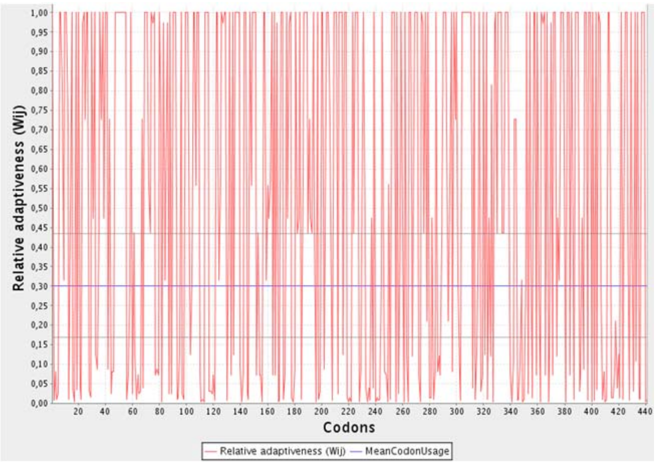

Fig. R1 Codon usage analysis of *xylA* for *C. glutamicum* using JCat.

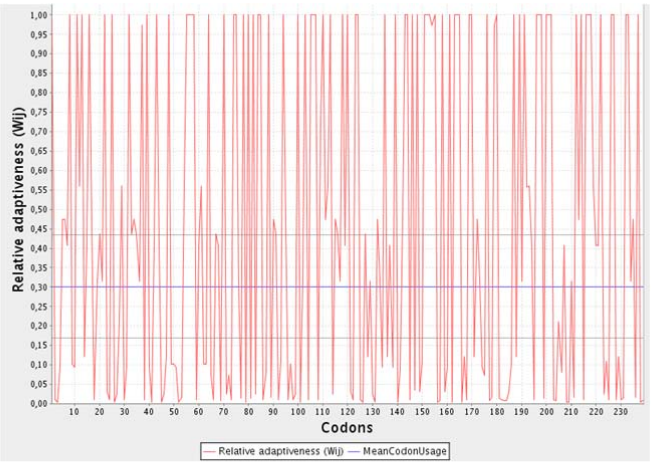

Fig. R2 Codon usage analysis of *gfp* for *C. glutamicum* using JCat.

4. Can author explain the rationale behind choosing eight consecutive G's as a low efficiency RBS using references?

Response

Thanks for your helpful comments. The RBS with eight consecutive Gs is used as the initial RBS is because it is easy to generate an A/G-rich RBS library. After measuring the GFP fluorescence controlled by this RBS, we found it is a low-efficiency RBS. We didn't find any reported study using such an RBS with eight consecutive Gs in literature. Therefore, we compared the strength of this RBS with several previously reported RBSs (Zhang, B. et al. Ribosome binding site libraries and pathway modules for shikimic acid synthesis with *Corynebacterium glutamicum*. Microb. Cell Fact. 14, 71 (2015)). The RBS GAAAGGCA CCCGAT is regarded as a low-efficiency RBS in this reference. However, with a *gfp* reporter, we found that the GGGGGGGG TTGAGA RBS used in this study was much weaker than GAAAGGCA CCCGAT. Therefore, GGGGGGGG TTGAGA was used as a low-efficiency RBS control. The data have been added as Supplementary Fig. 4.

Please refer to Supplementary Fig. 4:

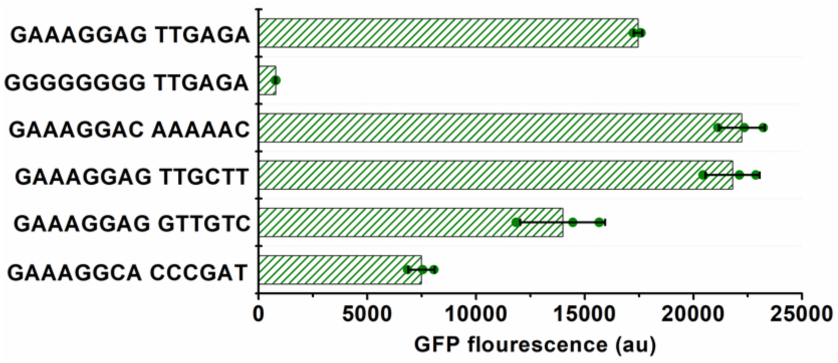

Supplementary Fig. 4 Strength comparison of the RBSs used in this study with previously reported RBSs. GAAAGGAG TTGAGA was used for expressing nCas9(D10A)-AID in plasmid pnCas9(D10A)-AID^{TS} and expressing *gfp* in *C. glutamicum* chromosome as the strong RBS control in Fig. 1d. GGGGGGGG TTGAGA was used as the tailored RBS for constructing RBS libraries using the BETTER method. GAAAGGAC AAAAAC, GAAAGGAG TTGCTT, GAAAGGAG GTTGTC were used as strong RBS controls, which were reported by Zhang et al[1]. GAAAGGCA CCCGAT was also reported by Zhang et al[1] and used for expressing nCas9(D10A)-AID in plasmid pnCas9(D10A)-AID-2^{TS}. RBS strength was determined using a chromosomal GFP reporter. Error bars indicate standard deviations from three parallel experiments.

5. In page 4, line 70 author claims "lack of efficient recombination pathways available in C. glutamicum". This argument is not entirely correct. Good progress has been made in recent years. Examples, Zhang, J., et al. Optimizing a CRISPR-Cpf1-based genome engineering system for Corynebacterium glutamicum. Liu, W, et al. Combined genome editing and transcriptional repression for metabolic pathway engineering in Corynebacterium glutamicum using a catalytically active Cas12a.

Response

Thanks for your helpful comments. After reading the two references you mentioned, we think they somewhat support our claim that the homologous recombination efficiency of *C. glutamicum* is low. These two studies did substantial work to optimize the CRISPR-Cpf1 (Cas12a)-based genome engineering methods for *C. glutamicum* because the homologous recombination efficiency of *C. glutamicum* is not as high as that of *Escherichia coli and Saccharomyces cerevisiae*. As shown in the Fig. 4d of

Zhang's paper, less than 100 *C. glutamicum* clones were obtained with the optimized CRISPR-Cpf1 (Cas12a)-based genome engineering, making multiplex editing of more than one target gene very difficult. In the discussion section of Liu's paper, the authors also claimed "However, due to the relative low homologous recombination efficiency of *C. glutamicum*, gene deletion efficiency was still lower than eukaryotes, such as yeast" and "Therefore, heterologous recombinase, such as RecT, should be introduced to further improve the genome editing efficiency of the RE-CRISPR system."

To make the statement more accurate and avoid ambiguity, we kept the "whose genetic manipulations suffer from low transformation and recombination efficiencies" but deleted "because of complex cell wall structure and lack of efficient recombination pathways" in the revised manuscript.

Please refer to Page 4, Line 75: "We successfully applied BETTER in industrially important and model microorganisms *Corynebacterium glutamicum* and *Bacillus subtilis*, whose genetic manipulations suffer from low transformation and recombination efficiencies[18-20] for large genetic combinatorial library creation."

6. There is no control used in Figure 2 (g) for xylose utilization. It will be good if author can show us the xylose utilization rate from the strain expressing xylA, tkt, xylB contains tailored RBS sequence, but without base editing. This could provide the expression variations between BETTER method vs direct evolution method in 6th passage.

Response

Thanks for your helpful comments. We have tested the growth of the strain expressing *xylA*, *xylB*, and *tkt* with tailored RBS sequence (GGGGGGGG) in minimal medium with xylose as the sole carbon source. However, even with six passages of serial cultivation, no cell growth was observed. This is possibly because the GGGGGGGG RBS is a very weak RBS and the expression levels of *xylA*, *xylB*, and *tkt* are too low to support cell growth on xylose. We added these data in Supplementary Fig. 5.

Please refer to Page 9, Line 183: "The starting strain without base editing was also serially cultivated as a control. After six passages of serial cultivation, growth of the edited cell mixture on xylose was significantly improved (Fig. 2c). However, the unedited starting strain did not grow on xylose (Supplementary Fig. 5), which was

possible due to the low-level expression of xylose utilizing enzymes with tailored GGGGGGGG RBS (Supplementary Fig. 6).”

Please refer to Supplementary Fig. 5:

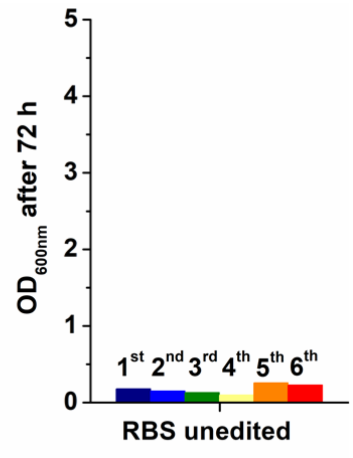

Supplementary Fig. 5 Serial cultivation of the starting strain with *xylA*, *xylB*, and *tkt* controlled by the tailored GGGGGGGG RBS on xylose. Numbers above columns represent the passages of serial cultivation.

7. In Fig. 2 (c) author stops at 6th passage? Will the OD600 be further improved after 6th passage? Will the dominant RBS variants remain the same after 6th passage?

Response

Thanks for your helpful comments. We conducted the serial cultivation for seven passages and found that the cell growth did not increase and the dominant RBS variants remained the same after six passages. Therefore, we used the culture of 6th passage to identify xylose-utilizing mutants and reported the growth and distribution of RBS variants of the first six passages. The distribution of RBS variants for the 7th passage is shown as follows.

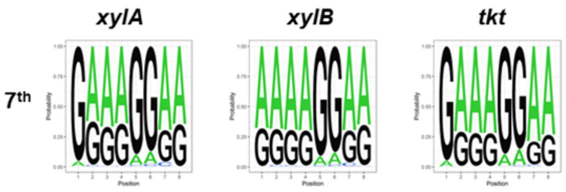

Fig. R3 Components of RBS libraries in the 7th passage of serial cultivation in xylose.

8. In Fig. 2 (g) the built strain growth declined after 20h but, the screened strain reaches stationary phase at 24h. Can author explain the possible reason for the difference?

Response

Thanks for your helpful comments. We noticed the difference between the growth of screened strain and built strain in stationary phase. At 20 h, xylose in the medium is exhausted and the cultures might reach the highest OD soon and then declined due to lack of carbon source. We took samples and detected OD and xylose every four hours and possibly missed the timing with the highest OD values (between 20-24 h). Therefore, some difference was observed for the last data point between the growth of screened strain and built strain. However, we think this doesn't affect the conclusion that the screened strain and built strain have similar cell growth and xylose utilization capability. Besides, we did a statistical analysis and the *P* value between the OD values of the screened strain and built strain is 0.066, suggesting no significant difference. We have added the statistical analysis in the revised Fig. 2g and its legend.

9. Can author reason out why the overexpression of dxr and ispD decreased lycopene titer? Will lycopene titer be improved if heterologous dxr and ispD introduced in the cells rather than altering native RBS region?

Response

Thanks for your helpful comments. We inserted a second copy of *dxr* and *ispD* into the *C. glutamicum* chromosome. Expression of the original copy is controlled by their native RBSs. During application of BETTER in regulating the expression of ten lycopene biosynthetic genes including *dxr* and *ispD*, the second copy of *dxr* and *ispD* in some strains such as No. 2, No. 3, and No. 4 is deleted from the chromosome, but the original copy still exists and is controlled by the native RBSs. We did not alter the native RBS regions of the original copy of *dxr* and *ispD*. We reasoned that too much expression of *dxr* and *ispD* (due to insertion of the second copy into chromosome) might reduce lycopene production and their original copy on *C. glutamicum* chromosome is sufficient. In this case, overexpression of *dxr* and *ispD* may produce excess proteins, overburden cells, or cause accumulation of toxic intermediates, which consequently inhibits lycopene biosynthesis. We have discussed the possible reason of this phenomenon in the revised manuscript.

Please refer to Page 16, Line 312: "For a biosynthetic pathway, proteins expressed at suboptimal levels could lead to problems, such as protein burden, accumulation of toxic intermediates, or metabolic imbalance[30]. It is speculated that overexpression of *dxr* and *ispD* might overburden the host and negatively affect lycopene biosynthesis in this case."

10. In page 18, line 355 replace the word from "detection" to "emission".

Response

Thanks for your helpful comments. We have changed the "detection" to "emission" in the revised manuscript.

11. RBS spelling error at Page 15, line 292.

Response

Thanks for your helpful comments. We have corrected this mistake in the revised manuscript.

Reviewer #2 (Remarks to the Author):

In this manuscript titled "In-situ generation of almost infinite genetic combinations for 3 metabolic reprogramming via CRISPR-guided base editing", the authors devised a targeted base-editing tool named BETTER and enabled rapid in situ generation of RBS libraries containing huge number of variants. Applying BETTER to Corynebacterium glutamicum, the authors generated strains utilizing xylose for growth or producing lycopene from glucose at noticeable performances, demonstrating great efficiency of this tool in generating RBS libraries. This manuscript was nicely written with well-designed experiments and thorough analysis of the experimental data. However, some numbers and expressions are exaggerated. In addition, more examples are expected to emphasize broad applicability of this efficient system. Additional set of experiments and rewording some sentences would upgrade the quality of this manuscript.

Response:

Thank you for your helpful comments. we did additional experiments and reworded some sentences according to your helpful suggestions. We hope the additional data supplemented in the revision can enable our manuscript to win your satisfaction.

Major comments

Title and manuscript: The title should be changed so that it reflects what they have actually done, rather than overly stating. Here, the authors have used nCas9-cystidine deaminase for the generation of large RBS libraries for up to 10 genes for the production of lycopene. It seems that this system may be useful only for the production of chemicals that can be screened using colorimetric assays. Also, for the authors to justify their title, they must examples of other genetic combinations other than RBS libraries. For example, they should have also shown promoter libraries, UTR libraries, terminator libraries, gene mutation libraries to encompass genetic combinations.

Response:

Thank you for your helpful comments. First, to justify the title, we did additional experiments to demonstrate the general applicability of this BETTER method in construction of RBS libraries, 5'UTR libraries, and promoter libraries in not only *C. glutamicum* but also another industrially important chassis and model microbe

Bacillus subtilis. Second, BETTER is an easy method to generate genetic combinations at multiple genomic sites. It can be used to improve the utilization of substrates, production of chemicals and enzymes, or any screenable phenotypes of many different microorganisms. Of course, a proper screening method, preferably a high-throughput one like growth-based enrichment, colorimetric assay, and FACS, is needed to screen the desired mutants from the large libraries. However, the screening method is independent from the BETTER method. In fact, all methods for generating mutations or genetic combinations need a good subsequent screening method. Therefore, we think the requirement of a screening method might not be considered as a limitation of the BETTER method.

The newly added data for 5'UTR and promoter editing are summarized as follows. Please refer to our response to your last major comment "Line 292-294" for the application of BETTER in *B. subtilis*.

Please refer to Page 16, Line 326: "**Application of BETTER in diversifying 5' untranslated region (5' UTR) and promoter.** Besides RBS, the promoter and the 5'UTR between RBS and the translational start codon also play important roles in controlling gene expression[26,27]. To expand the application of BETTER in diversifying other translation or transcription elements, we tested library construction for the 5'UTR between RBS and the start codon and the –35 box of promoter. The chromosomal *gfp* expression cassette in *C. glutamicum* was modified to change the original 5'UTR sequence from TTGAGA to six consecutive Cs, which could be edited by base editors to generate a C/T-rich library (Fig. 4a). Construction of a G/A-rich library initiated from consecutive Gs was not selected here to avoid its interference with the upstream RBS. Because there is no available NGG PAM downstream the target Cs, we introduced a synonymous mutation (GGA to GGG) to generate a GGG PAM. Because the target Cs do not locate at the editing window of Target-AID base editing tool (positions –20 to –16, counting the PAM as positions 1–3)[13], we used BE3[12] base editing tool with a larger editing window in *C. glutamicum* (positions –19 to –11)[32] to cover the target six Cs in 5'UTR (Fig. 4a). After base editing, a C/T-rich 5'UTR library was generated. Because an uracil DNA glycosylase inhibitor was included in BE3, few C to G and C to A conversions were observed (Fig. 4b). Although the C distal to PAM was less efficiently edited, all the 64 C/T-containing combinations were detected by NGS (Supplementary Table 6). The discreteness of 5'UTR variants generated by BETTER was evaluated using the KL

divergence analysis and a relatively low value of 1.98 was obtained (Supplementary Fig. 11). Totally 772 out of 4,096 kinds of G/A/C/T-containing combinations were detected (Supplementary Table 6). By analyzing GFP expression using flow cytometry, it was found that the edited cells showed diverse GFP fluorescence signals (Fig. 4b).

To regulate gene expression by editing promoter, the original –35 box of promoter P_{11F} used for *gfp* expression on *C. glutamicum* chromosome was changed from TGGCAA to six consecutive Cs. An available AGG PAM downstream the target Cs allowed direct BETTER application using Target-AID base editing tool (Fig. 4a). Base editing targeting the six Cs also generated all the 64 C/T-containing combinations with a KL divergence value of 1.95 (Supplementary Fig. 11). Totally 900 G/A/C/T-containing combinations were detected (Supplementary Table 6). The GFP fluorescence outputs of the edited cell cultures were diversified as expected (Fig. 4c). The results suggest that the BETTER method can also be used to diversify gene expression by generating libraries for other translation or transcription elements than RBS, such as 5'UTR and promoter."

Please refer to Fig. 4:

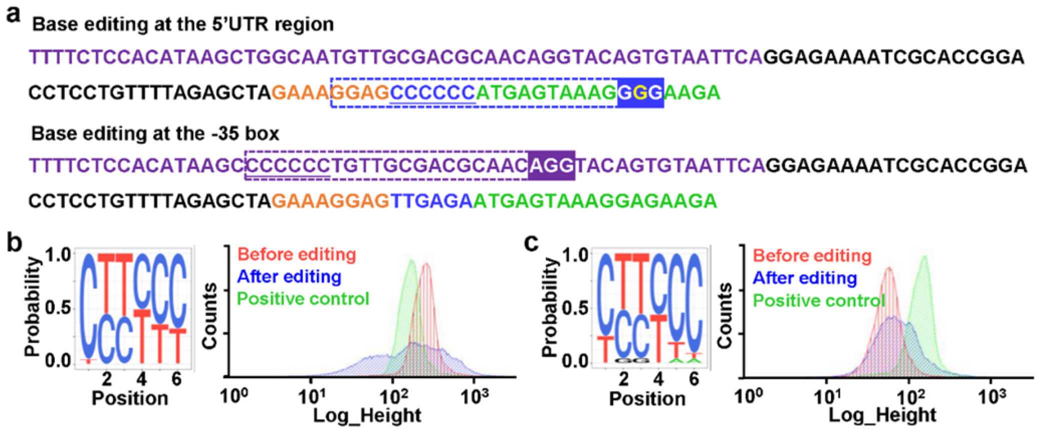

Fig. 4 Diversifying 5'UTR and promoter by BETTER for regulating gene expression. a, Design of base editing targets by replacing the original 5'UTR between RBS and start codon and –35 box of promoter to six consecutive Cs, respectively. The target Cs are underlined. The N20 sequences of gRNA are denoted with the dotted box. PAMs for 5'UTR and promoter editing are shaded in blue and purple, respectively. The mutated G for generation of a GGG PAM is highlighted in yellow. The original sequence for *gfp* expression cassette is shown Supplementary Fig. 1. **b**, **c**, Analyses of the 5'UTR (**b**) and –35 box (**c**) libraries by NGS and flow cytometry. Three clones were used for base editing and the cells were mixed with an equal

proportion before extraction of genomic DNAs, PCR amplification, and NGS. The original *gfp* expression cassette was used as a positive control.

Please refer to Supplementary Fig. 11:

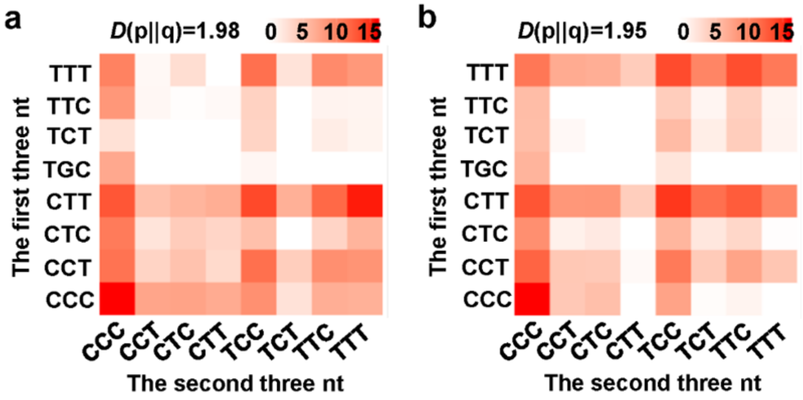

Supplementary Fig. 11 The matrixes showing the coverage and discreteness of 64 C/T-containing variants in the 5'UTR (**a**) and -35 box (**b**) libraries generated by BETTER. KL divergence ($D(p||q) = \sum_{i=1}^{n} p(x) log \frac{p(x)}{q(x)}$) was used to evaluate the discreteness of genetic combinations. Scale bar represents $\log_2$(relative proportion).

Throughout the manuscript: Some RBS sequences were mentioned strong or weak without appropriate data or references. Please provide evidences for their strength.

Response

Thanks for your helpful comments. The RBSs used in this study are from an RBS library reported by Zhang et al. (10.1186/s12934-015-0254-0) and another RBS library constructed by our lab. We have evaluated strength of these RBSs using the same GFP reporter and compared them with previously reported RBSs. The data have been added as Supplementary Fig. 4.

Please refer to Supplementary Fig. 4:

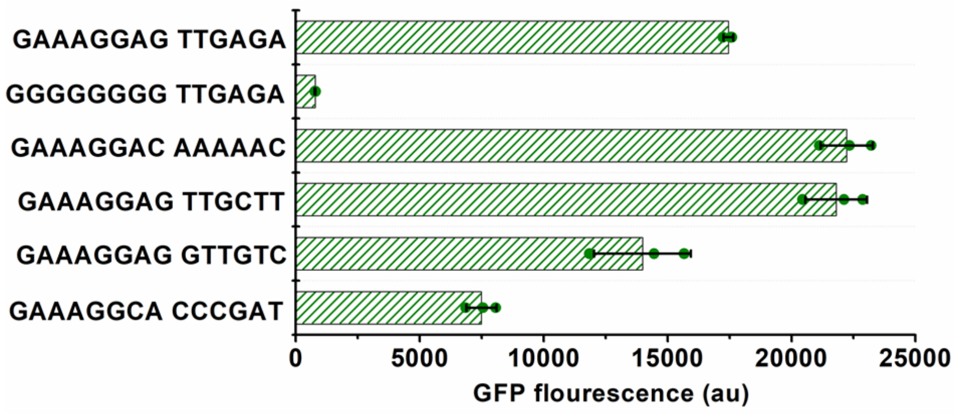

Supplementary Fig. 4 Strength comparison of the RBSs used in this study with previously reported RBSs. GAAAGGAG TTGAGA was used for expressing nCas9(D10A)-AID in plasmid pnCas9(D10A)-AID^{TS} and expressing *gfp* in *C. glutamicum* chromosome as the strong RBS control in Fig. 1d. GGGGGGGG TTGAGA was used as the tailored RBS for constructing RBS libraries using the BETTER method. GAAAGGAC AAAAAC, GAAAGGAG TTGCTT, GAAAGGAG GTTGTC were used as strong RBS controls, which were reported by Zhang et al[1]. GAAAGGCA CCCGAT was also reported by Zhang et al[1] and used for expressing nCas9(D10A)-AID in plasmid pnCas9(D10A)-AID-2^{TS}. RBS strength was determined using a chromosomal GFP reporter. Error bars indicate standard deviations from three parallel experiments.

Line 71-73: The distance between an RBS and a start codon affects the expression strength. However, the exact location of the tailored GGGGGGGG sequence before the RBS library generation was not mentioned. Please specify a clear rule of determining the location of GGGGGGGG introduction relative to the location of a start codon.

Response

Thanks for your helpful comments. We have added a supplementary figure (Supplementary Fig. 1) to clearly show the location and sequence of promoter, RBS, and *gfp* used in this study. According to a comprehensive analysis of the *C. glutamicum* transcriptome, the spacers between RBS and translational start codon are dominantly 5–10 nucleotides in *C. glutamicum* (BMC Genomics 14, 888 (2013)). Another study constructed a plasmid-borne RBS library with a seeding sequence of AAAGG(N)$_{6–9}$ATG and obtained gene expression at various levels, which verified the

previous transcriptome analysis. Therefore, a spacer of six nucleotides (TTGAGA) that has been experimentally verified for gene expression in *C. glutamicum* was inserted between the RBS and the start codon in this study.

To specify a clear rule of determining the location of GGGGGGGG introduction relative to the location of a start codon, we think it is best to follow the rules concluded from the transcriptome analysis, which is usually available for model or extensively studied microorganisms. However, for some non-model microorganisms without such analysis, our suggestion is to use experimentally verified RBS and spacer sequences and change the RBS to consecutive Gs. We have added this information in the revised "Methods" section.

Please refer to Page 26, Line 536: "Design of gene expression cassette. The gene expression cassettes for BETTER application in *C. glutamicum* and *B. subtilis* are shown in Supplementary Fig. 1 and Supplementary Fig. 12, respectively. Constitutive promoters P_{11F} and P_{gapDH} were used for gene transcription in *C. glutamicum* and *B. subtilis*, respectively[34,52]. The tailored GGGGGGGG RBS was inserted upstream of the target gene. To determine the location of introducing the tailored GGGGGGGG RBS relative to the location of a start codon, it is suggested to follow the rules concluded from the transcriptome analysis of the target host. For example, an analysis of *C. glutamicum* transcriptome suggests that the spacers between RBS and translational start codon are dominantly 5–10 nucleotides[53]. Therefore, a spacer of six nucleotides (TTGAGA) that has been experimentally verified for gene expression in *C. glutamicum* was inserted between the RBS and the start codon in this study. Such transcriptome analyses are usually available for model or extensively studied microorganisms. However, for some non-model microorganisms without such analyses, it is suggested to use experimentally verified RBS and spacer sequences and change the RBS to consecutive Gs for generation of RBS diversity using the BETTER method."

Line 81-82: Please provide total number of sequence reads examined to detect 94 and 202 variants, respectively. This information is critical to estimate the library-generating efficiency of BETTER.

Response

Thanks for your helpful comments. We have provided this information in the "Methods-NGS and data analysis" section. For all NGS analyses in this study, the

genomic DNAs of ~10^9 cells were extracted and used as templates to amplify the base editing region. The amplicon was sequenced and approximately 100,000 reads per sample were analyzed. We also added this information here.

Please refer to Page 5, Line 100: "After plasmid transformation and induced base editing, targeted next-generation sequencing (NGS) was used to examine the outcomes of base editing. For all NGS analyses, the genomic DNAs of ~10^9 cells were extracted and used as templates to amplify the base editing region. The amplicon was sequenced and approximately 100,000 reads per sample were analyzed."

Line 85-87: Please provide total number of reads examined to identify 527 and 970 G/A/C/T-containing RBS variants, respectively.

Response

Thanks for your helpful comments. Please refer to our response to your former comment. For all NGS analyses, the genomic DNAs of ~10^9 cells were extracted and used as templates to amplify the base editing region. The amplicon was sequenced and approximately 100,000 reads per sample were analyzed. This information has been added in both the "Results" and "Methods" sections.

Line 87-88: The expression "1,000-fold dynamic range of GFP expression" is ambiguous. Please consider rewording.

Response

Thanks for your helpful comments. We have reworded this sentence in the revised manuscript.

Please refer to Page 5, Line 116: "The strength of generated RBS variants spanned nearly three orders of magnitude in terms of GFP fluorescence intensity."

Line 116-118: Please explain the reason why 'the unexpected base editing before induction' did not occur while modifying the RBS of the gfp gene.

Response

Thanks for your helpful comments. We have rechecked the sanger sequencing data of base editing in *gfp* RBS and found the leaky editing before IPTG induction. As shown in the following figure, the leaky editing was not severe and did not lead to biased editing. Therefore, we did not notice it previously. We have added this information in the revised manuscript and supplementary material.

Please refer to Page 7, Line 146: "Prior to inducing the expression of CRISPR-guided cytidine deaminase, leaky base editing events severer than the single-site editing at RBS$_{gfp}$ were observed, which might be due to the increase in editing sites (Supplementary Fig. 3)."

Please refer to Supplementary Fig. 3:

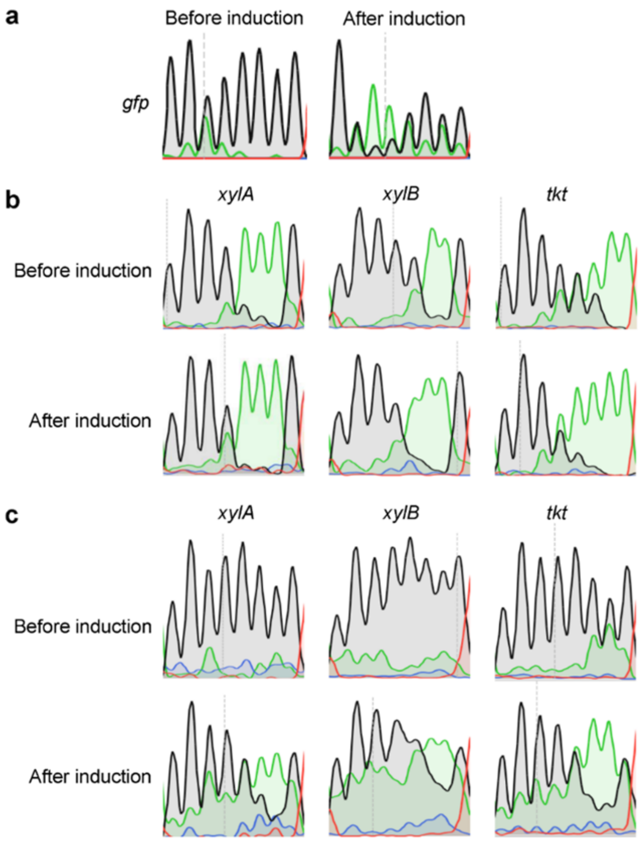

Supplementary Fig. 3 Analysis of base editing on the tailored GGGGGGGG RBS(s) of *gfp* and xylose utilization genes before and after IPTG induction by Sanger sequencing. a, Base editing using pnCas9(D10A)-AID^{TS} and pgRNA-RBS2. nCas9(D10A)-AID was expressed with a strong RBS (GAAAGGAGTTGAGA). Slightly leaky base editing events prior to induction were observed. **b,** Base editing using pnCas9(D10A)-AID^{TS} and pgRNA-RBS2. nCas9(D10A)-AID was expressed with a strong RBS (GAAAGGAGTTGAGA). Severer leaky base editing events prior to induction were observed, leading to bias editing of some nucleotides. **c,** Base editing using pnCas9(D10A)-AID-2^{TS} and pgRNA-RBS2. nCas9(D10A)-AID was expressed with a weaker RBS (GAAAGGCACCCGAT). Weakening translation of nCas9(D10A)-AID led to an overall moderate and average editing of eight Gs. Three clones were used for base editing and the cells were mixed with an equal proportion

before extraction of genomic DNAs, PCR amplification, and NGS. A direct comparison of the strength of used RBSs is shown in Supplementary Fig. 4.

Line 121-124: Again, please inform in this sentence the total number of variant population examined for each experimental set.

Response

Thanks for your helpful comments. Please refer to our response to your comment "Line 81-82". For all NGS analyses, the genomic DNAs of ~10^9 cells were extracted and used as templates to amplify the base editing region. The amplicon was sequenced and approximately 100,000 reads per sample were analyzed. This information has been added in both the "Results" and "Methods" sections.

Line 168-175: It has been well reported that the translation efficiency depends not on only on RBS sequence but also on the sequence of the 5' region of CDS. Thus, the enhancement of RBS strength measured using the GFP reporter system may not represent the actual RBS strength for the xylose utilization genes.

Response

Thanks for your helpful comments. It is true that the sequence of the 5' region of CDS affects translation efficiency. To measure the RBS strength for the xylose utilization genes in a more accurate way, we constructed a tailored reporter by fusing the first 180 bp of the target CDS (e.g. *xylA*) with *gfp*. A flexible linker sequence $(GGGGS)_3$ was added between these two parts to avoid the interference between them. However, this modification caused a large decrease in GFP fluorescence even with a strong RBS control, suggesting the GFP structure might be affected by the translation product of the 180 bp of the target CDS. Therefore, we didn't use this method to evaluate the RBS strength.

Instead, we directly measured the activities of xylose utilizing enzymes, which can also reflect the RBS strength for target gene expression. In the starting strain, *xylA*, *xylB*, and *tkt* were all controlled by the tailored GGGGGGGG RBS. In the screened strains, *xylA*, *xylB*, and *tkt* were controlled by RBSs GAAAGGAA, AAAAGGAA, and GAAAGGAA, respectively. As shown in Supplementary Fig. 6, the activities of XylA, XylB, ant Tkt in crude cell extracts increased by 7.0-, 5.8-, and 10.4-fold due to the RBS changes. The results also suggest that one RBS can lead to different expression levels for different target genes (eg. *gfp* and xylose catabolic genes)

possibly due to the 5' region of CDS. In this case, the BETTER method is highly preferred for optimizing gene expression since it helps to directly screen the optimal RBS for a target gene. We have added these data in the revised manuscript.

Please refer to Page 10, Line 211: "Since the 5' region of coding sequence (CDS) also effects gene expression[26,27], we directly measured activities of xylose utilizing enzymes in the starting strain with GGGGGGGG RBS and the screened strain with edited RBSs. The activities of XylA, XylB, ant Tkt in crude cell extracts increased by 7.0-, 5.8-, and 10.4-fold due to the RBS changes (Supplementary Fig. 6), which was intuitive and consistent with previous findings that xylose utilization benefited from the elevated expression of xylose catabolic genes[9]. The results also suggest that one RBS can lead to different expression levels for different target genes (eg. *gfp* and xylose catabolic genes) possibly due to the 5' region of CDS. In this case, the BETTER method is highly preferred for optimizing gene expression since it helps to directly screen the optimal RBS for a target gene."

Please refer to Supplementary Fig. 6:

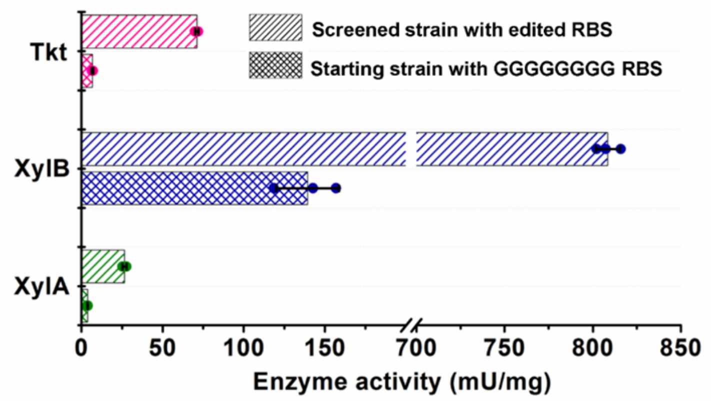

Supplementary Fig. 6 Activities of the xylose utilizing enzymes in the starting strain with GGGGGGGG RBS and the screened strain with edited RBSs. In the starting strain, *xylA*, *xylB*, and *tkt* were controlled by the tailored GGGGGGGG RBS. In the screened strains, *xylA*, *xylB*, and *tkt* were controlled by RBSs GAAAGGAA, AAAAGGAA, and GAAAGGAA, respectively. Error bars indicate standard deviations from three parallel experiments.

Line 229-231: 'Almost infinite' is an exaggeration since 2.7×10^{33} is definitely a finite number. In addition, the actual number of variants in the library was overestimated by simply multiplying library sizes of each gene – many combinations of variants

would not exist in the library unless the total cell population was much larger than 10^{33} (~10^5 mol of cells). Please provide the size of population examined to identify such a large number of diversity.

Response

Thanks for your helpful comments. We agree with you that such a large library size (2.7×10^{33}) cannot be obtained unless the total cell population was much larger than 10^{33} cells. For NGS in this study, the genomic DNAs of ~10^9 cells were extracted and used as templates to amplify the base editing region. The amplicon was sequenced and approximately 100,000 reads per sample were analyzed. Using current sequencing method, it is impossible to determine the exact size of the library. However, using the number of variants analyzed by sequencing, it is possible to estimate the theoretically maximum library size by multiplying library sizes of each gene. We have modified the description of library size in the revised manuscript to make it more rigorous. The NGS method has also been revised in the "Methods" section.

Please refer to Page 15, Line 279: "Targeted sequencing revealed the G/A/C/T-containing RBS types were 3,550 for *dxs* (including 237 G/A-containing types), 1,873 for *dxr* (including 221 G/A-containing types), 1,847 for *ispD* (including 238 G/A-containing types), 3,061 for *ispE* (including 237 G/A-containing types), 1,694 for *ispF* (including 237 G/A-containing types), 1,666 for *ispG* (including 223 G/A-containing types), 1,987 for *ispH* (including 236 G/A-containing types), 3,386 for *crtE* (including 245 G/A-containing types), 2,263 for *crtB* (including 236 G/A-containing types), and 1,667 for *crtI* (including 231 G/A-containing types) (Supplementary Table 4). It was estimated that the theoretically maximum library complexity could reach 2.7×10^{33} if a large enough population of cells were examined."

Line 233-235: Examination of only 10^5 cells out of a huge library is a big waste of resources. Examination of larger population with high-throughput screening devices would be helpful to find strains with better performances.

Response

Thanks for your helpful comments. We agree with you that screening of larger population will likely help us to obtain better lycopene producers. However, screening a better lycopene producer is not the main purpose of this study. The main novelty of

this study is that the BETTER method can easily create a large combinatorial library on multiple genomic sites. Besides, examination of a larger population than 10^5 cells will require a screening method with higher throughput than the used colorimetric screening method or other methods based on Raman spectroscopy or antioxidant capacity measurement (10.3390/s20133723; 10.1016/j.bej.2016.04.032). Flow cytometry would be a good choice. Therefore, we tested the possibility of screening lycopene producers from the library using flow cytometry. However, the signals of the intermediates of lycopene biosynthetic pathway interfere with the lycopene signal. A systematic optimization will be required prior to practical application of flow cytometry in lycopene producer screening. Therefore, we did not examine more cells to find a better lycopene producing strain.

Line 292-294: BETTER does seem so simple and efficient. The easiness and general applicability of BETTER would be greatly emphasized if additional examples in other microorganisms are included in this manuscript. Please apply BETTER to microorganisms other than C. glutamicum (e.g. Escherichia coli and Saccharomyces cerevisiae) and provide a couple of examples of improving strain performances. Otherwise, please confine the title to Corynebacterium glutamicum.

Response

Thanks for your helpful comments. We have applied BETTER in another microorganism *Bacillus subtilis* because it has wide applications in bioproduction of antibiotics, medicinal proteins, industrial enzymes, and biopolymers but is more difficult to genetically engineer than *Escherichia coli* and *Saccharomyces cerevisiae*. The newly added data are summarized as follows.

The newly added data have been added in the revised manuscript and are summarized as follows.

1. Application of BETTER in diversifying 5' untranslated region (5' UTR) and promoter.

To expand the application of BETTER in diversifying other translation or transcription elements, we tested library construction for the 5'UTR between RBS and the start codon and the –35 box of promoter. Starting from a CCCCCC 5'UTR or -35 box, BETTER generated a C/T-rich library. All the 64 C/T-containing combinations were detected by NGS. Totally 772 and 900 out of 4,096 kinds of G/A/C/T-containing combinations were detected for 5'UTR and -35 box, respectively (Supplementary

Table 6). By analyzing GFP expression using flow cytometry, it was found that the edited cells showed diverse GFP fluorescence signals (Figs. 4b and 4c). The results suggest that the BETTER method can also be used to diversify gene expression by generating libraries for other translation or transcription elements than RBS, such as 5'UTR and promoter.

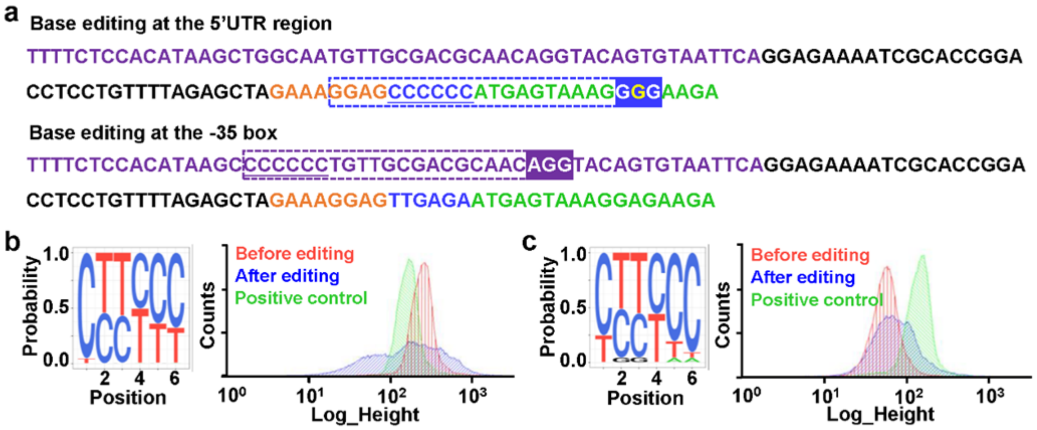

Fig. 4 Diversifying 5'UTR and promoter by BETTER for regulating gene expression. a, Design of base editing targets by replacing the original 5'UTR between RBS and start codon and –35 box of promoter to six consecutive Cs, respectively. The target Cs are underlined. The N20 sequences of gRNA are denoted with the dotted box. PAMs for 5'UTR and promoter editing are shaded in blue and purple, respectively. The mutated G for generation of a GGG PAM is highlighted in yellow. The original sequence for *gfp* expression cassette is shown Supplementary Fig. 1. **b**, **c**, Analyses of the 5'UTR (**b**) and –35 box (**c**) libraries by NGS and flow cytometry. Three colonies were used for base editing and the cells were mixed with an equal proportion before extraction of genomic DNAs, PCR amplification, and NGS. The original *gfp* expression cassette was used as a positive control.

2. Application of BETTER in *B. subtilis*.

In order to further verify the general applicability of the BETTER method for microorganisms other than *C. glutamicum*, the method was expanded to another model bacterium *B. subtilis*, in which we have recently developed an efficient base editing technology. GFP was first used as a reporter to test the BETTER method for construction of RBS library. The strength of generated G/A-rich RBS library spanned nearly three orders of magnitude in terms of GFP fluorescence intensity (Fig. 5b), demonstrating the good performance of BETTER in *B. subtilis*. A detailed analysis

reveals the generation of 230 out of 256 G/A-containing RBS variants with a KL divergence value of 1.87 (Fig. 5c), and the number increases to 3,519 in the case of calculating G/A/C/T-containing RBS variants (Supplementary Table 7).

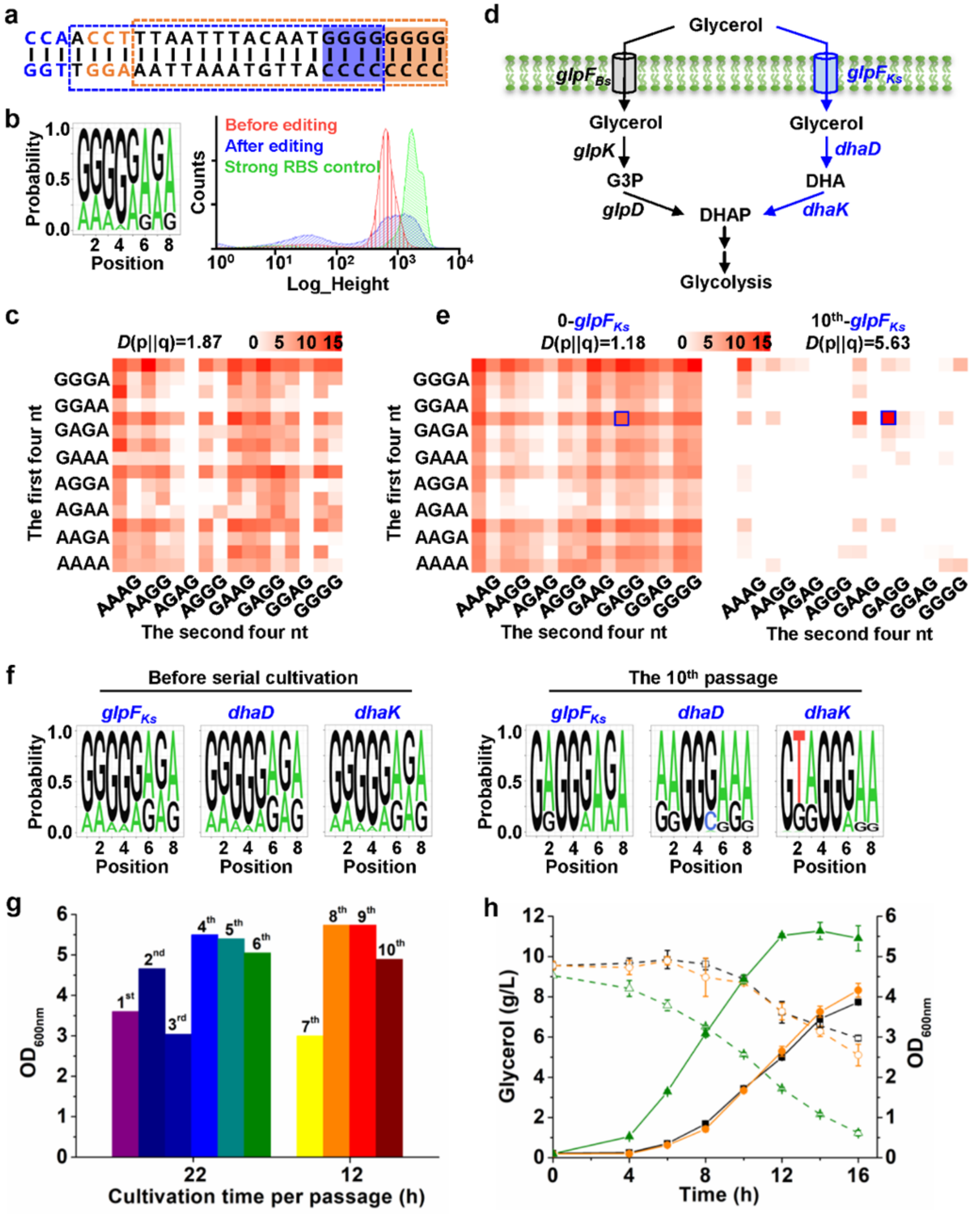

Fig. 5 Regulating *gfp* expression and reprogramming glycerol catabolism by BETTER in *B. subtilis*. a, Two interlaced gRNAs facilitate editing of eight Gs. A set of PAM, gRNA, and editing window is shown in the same color. **b**, Analyses of the RBS_{gfp} library by NGS and flow cytometry. Three colonies were used for base editing and the cells were mixed with an equal proportion before extraction of genomic

DNAs, PCR amplification, and NGS. A strong RBS (AGGAGGCG)[34] was used as a control. **c**, The matrix shows the coverage and discreteness of 256 G/A-containing RBS variants in the RBS_{gfp} library generated by BETTER with double gRNAs. Scale bar represents $\log_2$(relative proportion). KL divergence ($D(p \| q)$) was used to evaluate the discreteness of genetic combinations. **d**, The native glycerol catabolism pathway of *B. subtilis* (black arrows) and the heterologous pathway from *Klebsiella* sp. (blue arrows). G3P, glyceraldehyde 3-phosphate; DHA, dihydroxyacetone; DHAP, dihydroxyacetone phosphate. $glpF_{Bs}$, glycerol facilitator from *B. subtilis*; *glpK*, glycerol kinase; *glpD*, G3P dehydrogenase; $glpF_{Ks}$, glycerol facilitator from *Klebsiella* sp.; *dhaD*, glycerol dehydrogenase; *dhaK*, dihydroxyacetone kinase. **e**, The matrixes show the coverage and discreteness of 256 G/A-containing RBS variants in the RBS libraries of $glpF_{Ks}$ generated by BETTER before (0) and after ten passages (10th) of serial cultivation in glycerol. Scale bar represents $\log_2$(relative proportion). Blue squares represent the enriched RBS_{glpFKs} GAGGGAGA. The matrixes for RBS libraries of *dhaD* and *dhaK* are shown in Supplementary Fig. 14. Three colonies were used for base editing and the cells were mixed with an equal proportion before extraction of genomic DNAs, PCR amplification, and NGS. **f**, Components of RBS libraries before and after ten passages of serial cultivation in glycerol. **g**, Serial cultivation for screening genetic combinations that favor growth on glycerol. Numbers above columns represent the passages of serial cultivation. For the first six passages, transfer was made after 22 h cultivation. For the subsequent four passages, the cultivation time for each passage was reduced to 12 h because cell growth rate largely increased. **h**, Growth (filled symbols) and glycerol consumption (open symbols) of wild-type *B. subtilis* 168 (black square), the starting strain (orange circle) with heterologous $glpF_{Ks}$, *dhaD*, and *dhaK* genes controlled by GGGGGGGG RBSs, and the screened strain (green triangle) from the 10th passage with RBS_{glpFKs} GAGGGAGA, RBS_{dhaD} AAGGGAAA, and RBS_{dhaK} GTAGGGAA. Error bars indicate standard deviations from three parallel experiments.

Next, we reprogramed the glycerol catabolism pathway to accelerate glycerol utilization in *B. subtilis*. Glycerol is a major by-product of biodiesel production and a promising feedstock for biomanufacturing. It also enhances bioproduction of some valuable chemicals (e.g. menaquinone-7 and uridine) when used as the carbon source for *B. subtilis*. An artificial cluster consisting of a constitutive promoter P_{gapDH}, $glpF_{Ks}$

(encoding glycerol facilitator), *dhaD* (encoding glycerol dehydrogenase), and *dhaK* (encoding dihydroxyacetone kinase) from an efficient glycerol utilizer *Klebsiella* sp. was integrated into the *B. subtilis* 168 chromosome (Fig. 5d). BETTER generated good RBS libraries for three genes (Figs. 5e and 5f and Supplementary Table 8). The edited cell mixture was serially cultivated in M9 minimal medium with glycerol as a sole carbon source and accelerated growth rate was observed (Fig. 5g). NGS analysis shows that RBS_{glpFKs} GAGGGAGA, RBS_{dhaD} AAGGGAAA, and RBS_{dhaK} GTAGGGAA were enriched in varying degrees (Fig. 5f and Supplementary Fig. 14). The growth rate and glycerol uptake rate of the screened strain were apparently higher than those of the controls (Fig. 5h). The improvement demonstrates the applicability of BETTER in metabolic engineering of *B. subtilis*.

Minor comments

Line 28: Consider changing the verb form 'regulating' to 'regulate'.

Response

Thanks for your helpful comments. We have changed "regulating" to "regulate" in the revised manuscript.

Line 31: Suggest changing the word "shall" to "will".

Response

Thanks for your helpful comments. We have changed "shall" to "will" in the revised manuscript.

Line 37: 'burden' must be corrected into 'burdens'.

Response

Thanks for your helpful comments. We have corrected "burden" into "burdens" in the revised manuscript.

Line 54: 'notoriously' Suggest using a less subjective word.

Response

Thanks for your helpful comments. We have deleted this subjective word in the sentence.

Line 57-58: BETTER requires tailored RBS sequences with eight consecutive Gs, which requires DNA template-dependent engineering. Thus, "in a DNA template-free and DNA incorporation-independent manner" is incorrect. Please consider correcting the sentence.

Response

Thanks for your helpful comments. We have deleted this description in the sentence.

Please refer to Page 4, Line 72: "BETTER is designed to create complex libraries of genetic variants on multiple translation or transcription elements such as ribosome binding site (RBS) *in-situ* via base editing, which forms near-infinite genetic combinations for multiple genes expressed at diverse levels."

Line 163: Please provide quantitative kinetic data of the fastest xylose uptake rate in C. glutamicum to make the comparison easier.

Response

Thanks for your helpful comments. We have provided the fastest xylose uptake rate obtained in previous studies (5.7 mmol/gCDW·h) (Radek, A. et al. Bioresour. Technol. 2017, 245, 1377–1385) in the revised manuscript.

Line 174: Change 'elevated' to either 'the elevated' or 'an elevated'.

Response

Thanks for your helpful comments. We have changed "elevated" to "the elevated" in the revised manuscript.

Line 187-188: This sentence sounds weird. Please proofread this sentence.

Response

Thanks for your helpful comments. We have revised this sentence in the revised manuscript.

Please refer to Page 11, Line 235: "The artificial *dxs-dxr-ispD* and *ispE-ispF-ispG-ispH* clusters with tailored GGGGGGGG RBSs were inserted at the CGP2 prophage region and *upp* gene, respectively, as a second copy. The artificial *crtE-crtB-crtI* cluster with tailored GGGGGGGG RBSs was inserted between *cgl0622* and *cgl0627* to replace the native cluster (Fig. 3a).

Line 253: Please correct 'C. gultamicum' to 'C. glutamicum'

Response

Thanks for your helpful comments. We have corrected this mistake in the revised manuscript.

Line 255: Supplementary Fig. 5 should be cited instead of Supplementary Fig. 4.

Response

Thanks for your helpful comments. We have corrected this mistake in the revised manuscript. Because we have added some new supplementary figures in the revision, the former Supplementary Fig. 5 is now numbered as Supplementary Fig. 9.

Line 278: 'astonishing' Suggest using a less subjective word.

Response

Thanks for your helpful comments. We have changed "an astonishing number of" to "a large number of" in the revised manuscript.

Line 306: '.' is missing at the end of the sentence.

Response

Thanks for your helpful comments. We have corrected this mistake in the revised manuscript.

Reviewer #2 (Remarks to the Author):

Authors exerted much effort to address my comments, and improved their manuscript. I do not have any further comment.

Regarding the responses to the other reviewer's comments:
I think that this should be reviewed by Reviewer 1 whether the responses/modifications are satisfactory.
I nonetheless quickly went through the authors' responses and modifications made in the manuscript.
Authors addressed most of the comments.
One issue is "relatively low titer of lycopene compared with previous works", which was not improved.
I think that the reviewer 1 is concerned with the low yield even after all these efforts.
To me, the paper is ok as a tools and strategy paper.

Reviewer #2 (Remarks to the Author):

Authors exerted much effort to address my comments, and improved their manuscript.

I do not have any further comment.

Regarding the responses to the other reviewer's comments:

I think that this should be reviewed by Reviewer 1 whether the responses/modifications are satisfactory.

I nonetheless quickly went through the authors' responses and modifications made in the manuscript.

Authors addressed most of the comments.

One issue is "relatively low titer of lycopene compared with previous works", which was not improved.

I think that the reviewer 1 is concerned with the low yield even after all these efforts.

To me, the paper is ok as a tools and strategy paper.

Response:

Thank you for your helpful comments.
